# Fit-for-purpose curated database application in mass spectrometry-based targeted protein identification and validation

**DOI:** 10.1186/1756-0500-7-444

**Published:** 2014-07-10

**Authors:** Keding Cheng, Angela Sloan, Stuart McCorrister, Shawn Babiuk, Timothy R Bowden, Gehua Wang, J David Knox

**Affiliations:** 1 National Microbiology Laboratory, Public Health Agency of Canada, 1015 Arlington Street, Winnipeg, Manitoba R3E 3R2, Canada; 2 Department of Human Anatomy and Cell Sciences, Faculty of Medicine, University of Manitoba, 745 Bannatyne Avenue, Winnipeg, Manitoba R3E 0 J9, Canada; 3 National Centre for Foreign Animal Disease, Canadian Food Inspection Agency, 1015 Arlington Street, Winnipeg, Manitoba R3E 3R2, Canada; 4 Department of Immunology, Faculty of Medicine, University of Manitoba, 471 Apotex Centre, 750 McDermot Avenue, Winnipeg, MB R3E 0 T5, Canada; 5 Commonwealth Scientific and Industrial Research Organisation, Animal, Food and Health Sciences, Australian Animal Health Laboratory, Private Bag 24, Geelong, Victoria 3220, Australia; 6 Department of Medical Microbiology, Faculty of Medicine, University of Manitoba, 745 Bannatyne Avenue, Winnipeg, Manitoba R3E 0 J9, Canada

**Keywords:** Curated database, Targeted protein identification, Sheeppox virus, Flagellar typing, Tau, Recombinant prion protein

## Abstract

**Background:**

Mass spectrometry (MS) is a very sensitive and specific method for protein identification, biomarker discovery, and biomarker validation. Protein identification is commonly carried out by comparing MS data with public databases. However, with the development of high throughput and accurate genomic sequencing technology, public databases are being overwhelmed with new entries from different species every day. The application of these databases can also be problematic due to factors such as size, specificity, and unharmonized annotation of the molecules of interest. Current databases representing liquid chromatography-tandem mass spectrometry (LC-MS/MS)-based searches focus on enzyme digestion patterns and sequence information and consequently, important functional information can be missed within the search output. Protein variants displaying similar sequence homology can interfere with database identification when only certain homologues are examined. In addition, recombinant DNA technology can result in products that may not be accurately annotated in public databases. Curated databases, which focus on the molecule of interest with clearer functional annotation and sequence information, are necessary for accurate protein identification and validation. Here, four cases of curated database application have been explored and summarized.

**Findings:**

The four presented curated databases were constructed with clear goals regarding application and have proven very useful for targeted protein identification and biomarker application in different fields. They include a sheeppox virus database created for accurate identification of proteins with strong antigenicity, a custom database containing clearly annotated protein variants such as tau transcript variant 2 for accurate biomarker identification, a sheep-hamster chimeric prion protein (PrP) database constructed for assay development of prion diseases, and a custom *Escherichia coli* (*E. coli)* flagella (H antigen) database produced for MS-H, a new H-typing technique. Clearly annotating the proteins of interest was essential for highly accurate, specific, and sensitive sequence identification, and searching against public databases resulted in inaccurate identification of the sequence of interest, while combining the curated database with a public database reduced both the confidence and sequence coverage of the protein search.

**Conclusion:**

Curated protein sequence databases incorporating clear annotations are very useful for accurate protein identification and fit-for-purpose application through MS-based biomarker validation.

## Findings

The maturity of modern genomic sequencing technology has seen genomic databases being generated for more and more species and public databases growing larger every day. Owing to advanced instrumentation and powerful search engines, this mounting comprehensiveness and the refinement of databases have benefited mass spectrometry (MS)-based protein identification and biomarker discovery. However, despite improvement in these areas, MS-based protein characterization using public databases has not yet been perfected for all species. For instance, annotation of individual genes and their related protein products has not been standardized. As the setup of sequence-focused protein identification by MS is primarily based on post-proteolytic enzyme-digested peptides, much important annotation information, including the functions of proteins, can be ignored by the applied search engine [1]. It has been shown that search results can be optimized when using custom databases which focus on protein function with clear annotation, such as those generated using programs such as “Database on Demand” [1,2]. It has also been reported that search algorithms lose sensitivity when the search space (i.e. database size) is increased [3], and the more similar the database sequence to that of the protein of interest, the more accurate the search result [4]. These points are especially important during biomarker discovery and validation, as well as the protein identification of “non-mainstream” organisms [5]. Currently, many custom protein databases have been created to meet the special circumstances of the examined molecule, including prokaryotic ubiquitin-like protein (Pup) [6], proteins of O-GlcNAcylation [7], and a bio-molecular interaction network database [8].

In this paper, four projects spanning six years at the National Microbiology Laboratory in Canada, involving curated database creation and application for the purpose of biomarker identification and validation, are presented. All MS-based protein identification was performed using liquid chromatography tandem mass spectrometry (LC-MS/MS) detection and a Mascot database search algorithm. All the curated databases are presented in FASTA file format in Additional file 1. The detected proteins of interest are shown in Table 1.

**Table 1:** Search output produced by searching MS sequence data of various peptides against curated databases (CD) and the public databases, MSDB, NCBInr, and PBR

**Project**	**Sample source**	**Sample preparation**	**Targeted protein**	**Database: Top hit**			
				**Score**	**Peptide number**	**Score**	**Peptide number**
1^a^	Sheeppox virus	SDS-PAGE gel band	Unknown band (104 kD)	MSDB: lumpy disease virus protein		PBR: sheeppox virus protein	
				859	51	1039	80
2^b^	Human	In-solution digest	tau, transcript variant 2 (40.27 kD)	NCBInr: PNS specific tau, 78.8 kD		CD: tau, transcript variant 2, 40.27 kD	
				465	29 (17)¶	1615	34 (27)
3^b^	Sheep-hamster (chimera)	SDS-PAGE gel band	Sheep-hamster chimeric PrP	NCBInr: PrP in Dpc Micelles		CD: sheep-hamster chimeric PrP	
				4987	1(1)	3857	9(8)
4^b^	*E. coli*	In-solution digest	Flagellin H37	NCBInr: bacterial flagellin (*E. coli*)		CD: H37, gi|30059966|	
				18862	31(26)	29742	33(31)

^a^A QSTAR system was used to test the samples and Mascot database search with 0.4 kD peptide mass tolerance, 0.4 kD MS/MS tolerance, two missed tryptic cleavages, possible methionine oxidation, and all cysteine residues as carboxamidomethyl-cysteine due to alkylation with iodoacetamide.

^b^An Orbitrap system was used with 30 ppm peptide mass tolerance, 0.5 kD MS/MS tolerance, and two missed tryptic cleavage for all database searches. Oxidation on methionine and deamidation on glutamine and asparagines were chosen as possible modifications.

¶Numbers without brackets denote total specific peptide match numbers while numbers in brackets denote significant specific peptide match numbers as per the Mascot search engine.

The first project involved analyzing two SDS-PAGE (sodium dodecyl sulfate polyacrylamide gel electrophoresis) protein bands derived from sheeppox virus [9]. A western blot demonstrated that one protein band (“band A”) was immunologically very reactive to serum from sheep infected with the virus and, if identified, could have implications in vaccine design and/or reagent development for viral diagnoses. In-gel digestion was performed on this band, and LC-MS/MS implemented on the extracted tryptic peptides for peptide separation and detection. Mascot (Matrix Sciences) was used to perform the database search. When searching the public database, MSDB (Mass Spectrometry Sequence Database; 3,229,079 sequences; created by the Proteomics Group at Imperial College London), a protein identified as “putative virion core protein-lumpy skin disease virus” was identified with a Mascot score of 859 and a matched peptide number of 51. When searching the curated poxvirus specific database (21,000 sequences), created from the PBR (Poxvirus Bioinformatics Resource Centre) website (http://www.poxvirus.org/index.asp?bhcp=1), a more accurate identification was obtained (i.e. the “sheeppox virus protein”) with higher confidence (Mascot score = 1039) based on 80 peptide matches (Additional files 2 and 3). This observation clearly demonstrates that a smaller but more focused database is very useful for confirmation and validation of the molecule under study.

The second project employed MS to detect a protein with transcript variants. Microtubule-associated protein tau (or simply “tau”) has several variant forms [10,11]; examined in this study was tau transcript variant 2 (tau-2, GenBank accession NM_005910), routinely used in our laboratory as a biomarker for prion disease diagnosis [12]. When tau-2 MS data was searched against the public database, NCBInr (National Center for Biotechnology Information Non-Redundant), the “peripheral nervous system (PNS) specific tau” protein was primarily identified (Table 1, Additional file 4), when in fact tau-2 is a central nervous system tau variant. Moreover, top hits representing different variants of the same protein were obtained from searches using in-gel and in-solution digestions. These inconsistencies rendered quality control assessments of MS data difficult and consequently, a curated database with clear annotations was used to perform the search, where a consistent result was obtained (Table 1, Additional file 5).

In the third project, a curated database was employed to detect a protein that does not normally exist in nature. A recombinant sheep-hamster chimeric prion protein was designed for use in a novel and promising assay called “real-time quaking-induced conversion” (RT-QuIC), where low levels of infectious prion can be detected in human cerebral spinal fluid [13]. When the NCBInr database was used to confirm the existence of the chimeric protein from a digested SDS-PAGE band, only one peptide representing prion protein from different species (i.e. neither sheep nor hamster) was revealed (Table 1, Additional file 6), while the actual proteins [hamster (*Mesocricetus auratus*) and sheep (*Ovis aries*)] represented only the third and fourth hits, respectively. In order to accurately identify the chimeric protein, a curated database called “PrpSheep-Hamster” was created to accurately annotate and identify the protein (Table 1, Additional file 7). Indeed, database searches of MS data obtained from two separate but identical in-gel digested protein bands demonstrated that higher identification confidence and more sequence-specific peptide matches resulted from the smaller, more focused database (Table 2). This situation exemplifies that the characterization of proteins possessing rare tryptic enzyme digestion sites for MS analysis may benefit by using smaller and hence more accurate databases.

**Table 2:** Search output produced by searching sheep-hamster PrP MS sequence data against a curated prion protein database (CD) alone and in conjunction with the public database, Swissprot

**Sample**	**CD**^**a**^**only**		**CD and Swissprot**	
	**Mascot score**	**Peptide identified**	**Mascot score**	**Peptide identified**
SDS-PAGE gel band (replicate 1)	4117	12(11)¶	2232	12(10)
SDS-PAGE gel band (replicate 2)	2734	10(8)	1540	10(7)

¶Numbers without brackets denote total specific peptide match numbers while numbers in brackets denote significant specific peptide match numbers as per the Mascot search engine.

The fourth project highlights the ability of both MS and curated protein database to supplement traditional *E. coli* flagellar serotyping. As there are 53 flagellar serotypes in *E. coli* bacteria, serotyping by way of antigen-antibody agglutination reactions is a costly and tedious process [14,15]. In response to this, a unique method was developed to enrich flagella for high quality MS detection and identification [15], but problems arose when specific H types (i.e. serotypes) could not be obtained when searching the resulting MS data against the NCBInr database. Using the flagellar serotype H37, for example, a search of NCBInr listed the sequence as simply “flagellin” (Table 1, Additional file 8). To solve this problem, a curated *E. coli* flagellar database representing all serotypes was created as a FASTA file, using sequence data obtained from this public database of NCBInr. The custom database was used to successfully identify all examined flagella H types from reference *E. coli* strains [15] (Table 1 and Additional file 9 shows one example, H37). Searches using only the curated database, rather than using the curated and public database, Swissprot, in conjunction, also produced a larger number of matched peptides with higher confidence scores and often attained better coverage amidst shorter search times (Table 3). Lastly, MS sequence searches against the curated and public database, Swissprot and NCBInr, demonstrated that only the smaller, more focused curated database was able to obtain accurate top hit information with 100 % sensitivity and specificity (Table 4).

**Table 3:** **Search output produced by searching *E. coli *** **flagellin MS sequence data against a curated *E. coli *** **flagellin database (CD) alone and in conjunction with the public database, Swissprot**

**Strain number**	**Confirmed serotype**	**MS-H type**	**Mascot score**		**Sequence identified**		**Sequence coverage (%)**	
			**CD only**	**CD and Swissprot**	**CD only**	**CD and Swissprot**	**CD only**	**CD and Swissprot**
E169	H1	H1	14607	10922	57(55)¶	57(49)	98	98
E170	H2	H2	1754	1113	37(34)	37(27)	80	80
E171	H3	H3	8117	5735	52(46)	50(39)	91	90
E172	H4	H4	3894	2893	28(26)	28(21)	89	89
E173	H5	H5	1568	1167	26(23)	24(16)	81	74
E174	H6	H6	6123	4513	46(44)	46(38)	90	90
EDL933	H7	H7	6131	4511	56(54)	55(48)	90	90
E176	H8	H8	5538	3916	44(43)	43(39)	90	89
E177	H9	H9	10426	8099	53(51)	52(47)	80	80
E659	H10	H10	7281	5042	47(47)	47(41)	98	98
902380	H7	H7	3421	2515	43(40)	42(35)	84	82
050958	H7	H7	2656	1999	38(36)	38(31)	78	78
090414	H7	H7	5223	3943	46(44)	45(42)	94	94
091349	H7	H7	5887	4459	52(49)	52(46)	94	94
091350	H7	H7	3404	2522	44(42)	43(37)	89	88

¶Numbers without brackets denote total specific peptide match numbers while numbers in brackets denote significant specific peptide match numbers as per the Mascot search engine.

**Table 4:** **Top hits produced by searching *E. coli *** **flagellin MS data against a curated *E. coli *** **flagellin database (CD) and the public databases, Swiss-prot and NCBInr**^**a**^

**Strain number**	**Confirmed serotype**	**CD (195 sequences) top hit**	**Swiss-prot (331,337 sequen ces) top hit**	**NCBInr (25,303,445 sequences) top hit**
E169	H1	H1	*Shigella* flagellin	flagellin [*E. coli*]
E170	H2	H2	*E. coli* Elongation factor	flagellin [*E. coli*]
E171	H3	H3	*Salmonnella* flagellin	flagellin [*E. coli*]
E172	H4	H4	*E. coli* K12 flagellin	flagellin [*E. coli*]
E173	H5	H5	*E. coli* K12 flagellin	*E. coli* flagellar protein FliC
E174	H6	H6	*Shigella* flagellin	FliC [*E. Coli*]
EDL933	H7	H7	*Shigella* flagellin	flagellin [*E. coli*]
E176	H8	H8	*Shigella* flagellin	flagellin [*E. coli*]
E177	H9	H9	*Shigella* flagellin	flagellin [*E. coli*]
E659	H10	H10	*E. coli* K12 flagellin	flagellin [*E. coli*]
902380	H7	H7	*Shigella* flagellin	flagellin [*E. coli*]
050958	H7	H7	*Shigella* flagellin	flagellin [*E. coli*]
090414	H7	H7	*Shigella* flagellin	flagellin [*E. coli*]
091349	H7	H7	*Shigella* flagellin	flagellin [*E. coli*]
091350	H7	H7	*Shigella* flagellin	flagellin [*E. coli*]

^a^An Orbitrap system was used with 30 ppm peptide mass tolerance, 0.5 kD MS/MS tolerance, one missed tryptic cleavage for all database searches. Oxidation on methionine and deamidation on glutamine and asparagine were chosen as a possible modification.

## Conclusions

With the growing comprehensiveness of many species’ genomes and the maturity of MS-based technology, biomarker application and validation are being applied more and more for use in disease diagnosis and improvements of conventional bio-assay methods. From the above cases, it is evident that curated databases are very useful for accurate, specific, and consistent identification and confirmation of proteins and biomarkers of interest. Moreover, clearly annotated, fit-for-purpose databases prove extremely useful for high quality and standardized method development and validation using MS-based technology. Due to the sophistication of MS instrumentation and specific software requirements, together with variations in protein expression and posttranslational modifications, detection of analogous proteins through MS remains complicated. This paper will hopefully serve as an example and reminder for all MS users, especially those performing specific and/or “non-mainstream” research and applications, recombinant DNA technology quality control, and targeted biomarker identification and validation, to use curated fit-for-purpose databases in order to consistently and accurately identify MS data.

## Availability of supporting data

All the databases are available in the Additional file 1-Database.zip. Any questions regarding the application of the databases should be addressed to K. C. (Keding.Cheng@phac-aspc.gc.ca).

## Abbreviations

LC-MS/MS: Liquid-chromatography tandem mass spectrometry; MS: Mass spectrometry; MSDB: Mass spectrometry database; NCBInr: National Centre of Biotechnology Information Non-Redundant; PBR: Poxvirus Bioinformatics Resource Centre; PrP: Prion protein; Pup: Prokaryotic ubiquitin-like protein; RT-QuIC: Real-time quaking-induced conversion; SDS-PAGE: Sodium dodecyl sulfate polyacrylamide gel electrophoresis.

## Competing interests

The authors declare that they have no competing interests.

## Authors’ contributions

KC was responsible for preparing tryptic digest samples, collecting the data, and drafting the manuscript. AMS updated the *E. coli* flagella database and was responsible for critical writing of the manuscript. SM was responsible for mass spectrometry runs and database maintenance. SB was responsible for managing the poxvirus project and critical writing of the paper. TRB contributed to the design of the poxvirus project. GW was responsible for managing the *E. coli* flagellar typing project. JKD was responsible for managing the tau-2 and prion protein detection project, as well as critical writing of the paper. All authors read and approved the final manuscript.

## Supplementary Material

Additional file 1 Contains four individual databases in FASTA file format. Click here for file

Additional file 2 Sheeppox Virus-band A-MSDB search. Click here for file

Additional file 3 Sheeppox Virus-band A-PBRdb search. Click here for file

Additional file 4 Tau-2 NCBInr DB search. Click here for file

Additional file 5 Tau-2 custom DB search. Click here for file

Additional file 6 Sh-Ha Chimeric PrP-NCBInr DB search. Click here for file

Additional file 7 Sh-Ha PrP-custom DB search. Click here for file

Additional file 8 H37-NCBInr DB search. Click here for file

Additional file 9 H37-E coli-flagellar DB search. Click here for file
