# Supplementary material for: Fit-for-purpose curated database application in mass spectrometry-based targeted protein identification and validation
Source: BMC Res Notes. 2014 Jul 10;7:444. doi: 10.1186/1756-0500-7-444 (PMC4102332; doi:10.1186/1756-0500-7-444)
Supplement: Additional file 2 — Sheeppox Virus-band A-MSDB search. [file 1756-0500-7-444-S2.pdf]

Mascot Search Results

```
Search title     :
MS data file     : L:\Client Info\Kitching\Data\20061017-PK-GP\20061101-02 20061017-PK-GP band A.mgf
Database         : MSDB 20060831 (3239079 sequences; 1079594700 residues)
Timestamp        : 22 Nov 2006 at 14:51:53 GMT
Significant hits: Q8JTS2_LSDV   Putative virion core protein.- Lumpy skin disease virus (LSDV).
                  Q77GJ8_LSDV   Putative virion core protein.- Lumpy skin disease virus NW-LW.
                  K2C1_HUMAN    Keratin, type II cytoskeletal 1 (Cytokeratin-1) (CK-1) (Keratin-1) (K1) (67 kDa cytokeratin) (Hair
                  KRHU2         keratin 1, type II, cytoskeletal - human
                  TRPGTR        trypsin (EC 3.4.21.4) precursor - pig (tentative sequence)
                  Q8JTT4_LSDV   Putative early transcription factor small subunit.- Lumpy skin disease virus (LSDV).
                  Q4VAQ2_HUMAN  Keratin 2A (Epidermal ichthyosis bullosa of Siemens).- Homo sapiens (Human).
                  Q6IG03_RAT    Type II keratin Kb36.- Rattus norvegicus (Rat).
                  Q77GM4_LSDV   Putative DNA-binding virion core protein.- Lumpy skin disease virus NW-LW.
                  KRBOVI        keratin, 54K type I cytoskeletal - bovine
                  Q9DHL2_YLDV   101L protein.- Yaba-like disease virus (YLDV).
                  Q6PVZ3_CHICK  Type II alpha-keratin IIC.- Gallus gallus (Chicken).
                  Q90ZF7_RANCA  Keratin 8.- Rana catesbeiana (Bull frog).
                  K2C8_MOUSE    Keratin, type II cytoskeletal 8 (Cytokeratin-8) (CK-8) (Keratin-8) (K8) (Cytokeratin endo A).- Mus
                  T08212        RNA-directed RNA polymerase (EC 2.7.7.48) - Hendra virus
```

Probability Based Mowse Score

Ions score is -10*Log(P), where P is the probability that the observed match is a random event.
Individual ions scores > 48 indicate identity or extensive homology (p<0.05).
Protein scores are derived from ions scores as a non-probabilistic basis for ranking protein hits.

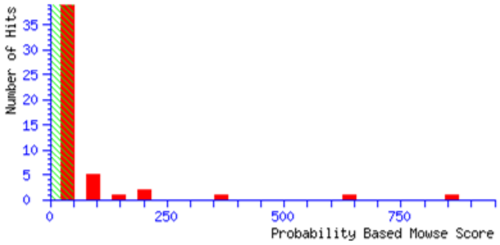

Peptide Summary Report

Format As: Peptide Summary — Help

Significance threshold p< 0.05 — Max. number of hits 20

Standard scoring (selected) — MudPIT scoring — Ions score cut-off 0 — Show sub-sets

Show pop-ups (selected) — Suppress pop-ups — Sort unassigned: Decreasing Score — Require bold red

Select All — Select None — Search Selected — Error tolerant — Archive Report

1. Q8JTS2_LSDV **Mass:** 104651 **Score:** 859 **Queries matched:** 51
Putative virion core protein.- Lumpy skin disease virus (LSDV).
Check to include this hit in error tolerant search or archive report

	Query	Observed	Mr(expt)	Mr(calc)	Delta	Miss	Score	Expect	Rank	Peptide
	9	387.2242	772.4338	772.4330	0.0008	0	24	31	3	K.SPELSIK.E
	16	405.2216	808.4286	808.4079	0.0207	0	10	4.8e+02	5	K.YVDISGR.D
[x]	29	412.7236	823.4327	823.4262	0.0065	0	27	8.5	1	R.ELLMYR.L
[x]	31	414.7536	827.4926	827.4865	0.0062	0	34	2	1	R.LNNLNIK.Y
	43	438.2365	874.4585	874.4548	0.0036	0	36	1.7	3	R.FQDQIPK.L
[x]	75	531.7860	1061.5574	1061.5505	0.0069	0	(28)	8.4	1	R.DFENLLGVR.R
[x]	76	531.7903	1061.5660	1061.5505	0.0155	0	28	7.9	1	R.DFENLLGVR.R
[x]	77	531.7946	1061.5747	1061.5505	0.0242	0	(18)	97	1	R.DFENLLGVR.R
[x]	99	568.8259	1135.6372	1135.6311	0.0062	0	(40)	0.44	1	K.EVLMLYAGLK.I
	100	568.8304	1135.6462	1135.6311	0.0151	0	(11)	3.4e+02	2	K.EVLMLYAGLK.I
[x]	104	576.8200	1151.6254	1151.6260	-0.0006	0	44	0.17	1	K.EVLMLYAGLK.I + Oxidation (M)
	113	585.2987	1168.5828	1168.5876	-0.0048	1	(29)	6.6	4	R.LKYPEYNSR.F
[x]	114	585.3055	1168.5964	1168.5876	0.0088	1	40	0.64	1	R.LKYPEYNSR.F
[x]	131	403.5504	1207.6293	1207.6197	0.0097	0	41	0.39	1	K.IDDNGSPHLIK.K
[x]	140	611.8180	1221.6214	1221.6353	-0.0139	0	(33)	2.5	1	K.DVLSTSIQGFR.V
[x]	141	611.8226	1221.6307	1221.6353	-0.0046	0	(21)	37	1	K.DVLSTSIQGFR.V
[x]	142	611.8250	1221.6354	1221.6353	0.0000	0	37	0.92	1	K.DVLSTSIQGFR.V
[x]	143	611.8250	1221.6354	1221.6353	0.0000	0	(24)	20	1	K.DVLSTSIQGFR.V
	144	611.8296	1221.6446	1221.6353	0.0093	0	(13)	2.4e+02	2	K.DVLSTSIQGFR.V
[x]	145	611.8319	1221.6493	1221.6353	0.0140	0	(16)	1.2e+02	1	K.DVLSTSIQGFR.V

	Query	Observed	Mr(expt)	Mr(calc)	Delta	Miss	Score	Expect	Rank	Peptide
☑	146	611.8342	1221.6539	1221.6353	0.0186	0	(25)	15	1	K.DVLSTSIQGFR.V
☑	147	611.8389	1221.6632	1221.6353	0.0279	0	(23)	21	1	K.DVLSTSIQGFR.V
☑	167	636.2887	1270.5629	1270.5506	0.0124	0	(18)	1e+02	1	R.NYQEYYYTK.N
☑	168	636.2911	1270.5677	1270.5506	0.0171	0	18	93	1	R.NYQEYYYTK.N
☑	209	678.8099	1355.6052	1355.6135	-0.0083	0	73	0.00023	1	R.AMDTDILTMSMK.H
☑	220	686.8148	1371.6150	1371.6084	0.0066	0	(40)	0.51	1	R.AMDTDILTMSMK.H + Oxidation (M)
☑	223	694.8146	1387.6147	1387.6033	0.0114	0	(38)	0.74	1	R.AMDTDILTMSMK.H + 2 Oxidation (M)
☑	320	795.4419	1588.8693	1588.8613	0.0080	0	70	0.00036	1	R.DHYYINLLNLLAK.E
☑	321	530.6315	1588.8728	1588.8613	0.0115	0	(52)	0.025	1	R.DHYYINLLNLLAK.E
☑	322	530.6315	1588.8728	1588.8613	0.0115	0	(49)	0.052	1	R.DHYYINLLNLLAK.E
☑	323	530.6380	1588.8923	1588.8613	0.0310	0	(50)	0.035	1	R.DHYYINLLNLLAK.E
☑	331	799.9269	1597.8393	1597.8351	0.0042	0	58	0.0065	1	K.LGINYLLDVYSSNK.L
	334	804.4405	1606.8664	1606.8640	0.0024	0	20	42	2	K.VISSILPSLCLDYK.V
☑	395	588.6642	1762.9709	1762.9802	-0.0093	0	43	0.17	1	K.SIIGSNNHTIISVRPR.V
☑	396	888.9325	1775.8505	1775.8406	0.0099	0	27	7.5	1	K.LSLFWDGIDYQEYK.S
	435	663.3000	1986.8782	1986.9839	-0.1057	0	(13)	2.1e+02	2	K.TSYVHPFDVLLHPDYGK.I
☑	436	663.3273	1986.9601	1986.9839	-0.0238	0	62	0.0028	1	K.TSYVHPFDVLLHPDYGK.I
☑	437	663.3418	1987.0036	1986.9839	0.0197	0	(19)	51	1	K.TSYVHPFDVLLHPDYGK.I
☑	438	663.3442	1987.0108	1986.9839	0.0269	0	(16)	1.2e+02	1	K.TSYVHPFDVLLHPDYGK.I
☑	439	663.3539	1987.0398	1986.9839	0.0559	0	(33)	2.3	1	K.TSYVHPFDVLLHPDYGK.I
☑	461	713.0257	2136.0553	2136.1367	-0.0814	0	(26)	7.5	1	K.LFHNVINPGYITPNDVVPK.F
☑	462	713.0432	2136.1079	2136.1367	-0.0288	0	(7)	6.1e+02	1	K.LFHNVINPGYITPNDVVPK.F
☑	463	713.0508	2136.1305	2136.1367	-0.0062	0	51	0.022	1	K.LFHNVINPGYITPNDVVPK.F
☑	464	713.0533	2136.1380	2136.1367	0.0013	0	(33)	1.5	1	K.LFHNVINPGYITPNDVVPK.F
☑	465	713.0558	2136.1455	2136.1367	0.0088	0	(22)	15	1	K.LFHNVINPGYITPNDVVPK.F
☑	466	713.0583	2136.1530	2136.1367	0.0163	0	(36)	0.71	1	K.LFHNVINPGYITPNDVVPK.F
☑	475	720.0307	2157.0704	2157.0564	0.0140	0	34	1.2	1	K.LISASYDLLHFMVSAGDYR.N
☑	481	733.0410	2196.1011	2196.1102	-0.0091	0	48	0.047	1	R.FVGGYVKPVEGGFDIQVEEK.I
☑	525	1229.6131	2457.2116	2457.1807	0.0309	0	36	0.65	1	-.MMPINAVTTLDQLEDSEYLFK.V
☑	529	1237.6021	2473.1896	2473.1756	0.0139	0	(21)	20	1	-.MMPINAVTTLDQLEDSEYLFK.V + Oxidation (M)
☑	530	1245.5970	2489.1795	2489.1705	0.0089	0	(29)	3.1	1	-.MMPINAVTTLDQLEDSEYLFK.V + 2 Oxidation (M)

Proteins matching the same set of peptides:
Q91MR2_LSDV **Mass:** 104665 **Score:** 859 **Queries matched:** 51
LSDV101 putative virion core protein.- Lumpy skin disease virus NI-2490.

2. Q77GJ8_LSDV **Mass:** 76229 **Score:** 660 **Queries matched:** 26
Putative virion core protein.- Lumpy skin disease virus NW-LW.
☐ Check to include this hit in error tolerant search or archive report

	Query	Observed	Mr(expt)	Mr(calc)	Delta	Miss	Score	Expect	Rank	Peptide
☑	52	464.2518	926.4890	926.5007	-0.0118	0	32	3	1	R.ANIHTMIK.K
☑	68	521.7648	1041.5151	1041.5130	0.0020	0	52	0.032	1	K.GEGASFFLSK.Q
	69	521.7648	1041.5151	1041.5130	0.0020	0	(9)	6.8e+02	2	K.GEGASFFLSK.Q
☑	72	530.8087	1059.6029	1059.5924	0.0105	0	49	0.068	1	K.QEILLSTTR.F
☑	101	570.7783	1139.5420	1139.5492	-0.0072	0	32	3	1	K.SSLTNSSMSVK.S
☑	112	583.7816	1165.5486	1165.5471	0.0015	0	45	0.13	1	K.MEEAMSLISR.Q
☑	119	591.7884	1181.5623	1181.5420	0.0203	0	(27)	9.5	1	K.MEEAMSLISR.Q + Oxidation (M)
☑	187	656.8641	1311.7136	1311.7186	-0.0051	0	(44)	0.2	1	R.NELFELLAHVK.S
☑	188	656.8713	1311.7280	1311.7186	0.0094	0	(38)	0.8	1	R.NELFELLAHVK.S
☑	189	656.8761	1311.7376	1311.7186	0.0190	0	44	0.19	1	R.NELFELLAHVK.S
☑	244	724.3739	1446.7332	1446.7289	0.0044	0	51	0.034	1	K.VNPDNYMLLVNR.L
☑	245	724.3890	1446.7635	1446.7289	0.0347	0	(16)	1e+02	1	K.VNPDNYMLLVNR.L
☑	253	732.3732	1462.7319	1462.7238	0.0081	0	(40)	0.42	1	K.VNPDNYMLLVNR.L + Oxidation (M)
☑	316	525.9468	1574.8186	1574.8238	-0.0052	1	41	0.3	1	K.KVNPDNYMLLVNR.L
☑	325	531.2821	1590.8245	1590.8188	0.0057	1	(32)	2.3	1	K.KVNPDNYMLLVNR.L + Oxidation (M)
☑	367	557.3079	1668.9020	1668.8947	0.0073	1	31	2.9	1	R.SNRNELFELLAHVK.S
☑	369	558.6573	1672.9500	1672.9161	0.0338	0	(24)	15	1	K.STHPLVLIHSHAHPK.I
☑	370	558.6595	1672.9566	1672.9161	0.0405	0	32	2.5	1	K.STHPLVLIHSHAHPK.I
☑	455	698.3349	2091.9829	2091.9791	0.0037	0	30	2.8	1	K.QQMLMNHITMFDDDLLK.M
☑	456	1051.9661	2101.9177	2101.9228	-0.0052	0	61	0.002	1	K.TSNNNQLDESDFYEWLK.G
☑	457	703.6693	2107.9860	2107.9740	0.0119	0	(18)	44	1	K.QQMLMNHITMFDDDLLK.M + Oxidation (M)
☑	459	709.0040	2123.9901	2123.9690	0.0211	0	(24)	11	1	K.QQMLMNHITMFDDDLLK.M + 2 Oxidation (M)
☑	491	744.3471	2230.0194	2230.0178	0.0016	1	45	0.087	1	R.KTSNNNQLDESDFYEWLK.G
☑	513	594.2805	2373.0928	2373.0848	0.0080	0	62	0.0014	1	R.FQSIHFVDMSSSSDLAFHYR.D
☑	515	598.2795	2389.0891	2389.0797	0.0094	0	(61)	0.0019	1	R.FQSIHFVDMSSSSDLAFHYR.D + Oxidation (M)
☑	518	1209.6478	2417.2811	2417.2689	0.0122	0	54	0.0087	1	R.LTEEAPIVFTGISDVISTEIQR.A

3. K2C1_HUMAN **Mass:** 66018 **Score:** 365 **Queries matched:** 8
Keratin, type II cytoskeletal 1 (Cytokeratin-1) (CK-1) (Keratin-1) (K1) (67 kDa cytokeratin) (Hair
☐ Check to include this hit in error tolerant search or archive report

Query	Observed	Mr(expt)	Mr(calc)	Delta	Miss	Score	Expect	Rank	Peptide
179	650.7722	1299.5298	1299.5223	0.0075	0	14	1.6e+02	2	K.NMQDMVEDYR.N
181	651.8637	1301.7129	1301.7078	0.0051	0	69	0.00065	1	R.SLDLDSIIAEVK.A
210	679.3525	1356.6904	1356.6885	0.0020	0	62	0.0029	1	K.LNDLEDALQQAK.E
222	692.3469	1382.6792	1382.6830	-0.0038	0	56	0.01	1	K.SLNNQFASFIDK.V
260	738.3990	1474.7834	1474.7780	0.0055	0	41	0.33	1	R.FLEQQNQVLQTK.W
356	546.9624	1637.8655	1637.8525	0.0130	1	35	1.1	1	K.SLNNQFASFIDKVR.F
386	858.9312	1715.8478	1715.8438	0.0040	0	54	0.015	1	K.QISNLQQSISDAEQR.G
554	1104.8028	3311.3865	3311.3006	0.0859	0	36	0.16	1	R.GSYGSGGSSYGSGGGSYGSGGGGGGHGSYGSGSSSGGYR.G

Proteins matching the same set of peptides:

AAG41947 **Mass:** 66198 **Score:** 365 **Queries matched:** 8
AF304164 NID: - Homo sapiens

AAF60327 **Mass:** 66149 **Score:** 365 **Queries matched:** 8
AF237621 NID: - Homo sapiens

---

4. KRHU2 **Mass:** 65569 **Score:** 205 **Queries matched:** 5
keratin 1, type II, cytoskeletal - human
Check to include this hit in error tolerant search or archive report

Query	Observed	Mr(expt)	Mr(calc)	Delta	Miss	Score	Expect	Rank	Peptide
179	650.7722	1299.5298	1299.5223	0.0075	0	14	1.6e+02	2	K.NMQDMVEDYR.N
210	679.3525	1356.6904	1356.6885	0.0020	0	62	0.0029	1	K.LNDLEDALQQAK.E
260	738.3990	1474.7834	1474.7780	0.0055	0	41	0.33	1	R.FLEQQNQVLQTK.W
386	858.9312	1715.8478	1715.8438	0.0040	0	54	0.015	1	K.QISNLQQSISDAEQR.G
554	1104.8028	3311.3865	3311.3006	0.0859	0	35	0.2	2	R.GSYGSGGGSYGSGGSSYGSGGGGGGHGSYGSGSSSGGYR.G

---

5. TRPGTR **Mass:** 25078 **Score:** 185 **Queries matched:** 3
trypsin (EC 3.4.21.4) precursor - pig (tentative sequence)
Check to include this hit in error tolerant search or archive report

Query	Observed	Mr(expt)	Mr(calc)	Delta	Miss	Score	Expect	Rank	Peptide
36	421.7629	841.5113	841.5021	0.0092	0	55	0.017	1	R.VATVSLPR.S
70	523.2807	1044.5468	1044.5564	-0.0095	0	42	0.36	1	K.LSSPATLNSR.V
487	737.7059	2210.0958	2210.0967	-0.0009	0	88	4.6e-06	1	R.LGEHNIDVLEGNEQFINAAK.I

Proteins matching the same set of peptides:

1AKSA **Mass:** 13569 **Score:** 185 **Queries matched:** 3
alpha trypsin (EC 3.4.21.4), chain A - pig

1AN1E **Mass:** 24142 **Score:** 185 **Queries matched:** 3
tryptase inhibitor, chain E - medicinal leech (fragments)

1AVWA **Mass:** 24145 **Score:** 185 **Queries matched:** 3
trypsin (EC 3.4.21.4), chain A - pig

1D3OA **Mass:** 24143 **Score:** 185 **Queries matched:** 3
trypsin (EC 3.4.21.4), chain A - pig

1EPTB **Mass:** 8928 **Score:** 185 **Queries matched:** 3
Porcine e-trypsin (EC 3.4.21.4), chain B - pig

---

6. Q8JTT4_LSDV **Mass:** 73485 **Score:** 137 **Queries matched:** 3
Putative early transcription factor small subunit.- Lumpy skin disease virus (LSDV).
Check to include this hit in error tolerant search or archive report

Query	Observed	Mr(expt)	Mr(calc)	Delta	Miss	Score	Expect	Rank	Peptide
203	669.8513	1337.6881	1337.6867	0.0015	0	61	0.0038	1	R.YNDEIITVPFK.L
227	699.9470	1397.8794	1397.8646	0.0148	0	28	5.2	1	K.VYILVPNINILK.I
364	830.4257	1658.8369	1658.8403	-0.0034	0	48	0.08	1	R.IYSILESISENYTK.E

Proteins matching the same set of peptides:

Q91MS8_LSDV **Mass:** 73500 **Score:** 137 **Queries matched:** 3
LSDV084 putative early transcription factor small subunit.- Lumpy skin disease virus NI-2490.

---

7. Q4VAQ2_HUMAN **Mass:** 65678 **Score:** 102 **Queries matched:** 2
Keratin 2A (Epidermal ichthyosis bullosa of Siemens).- Homo sapiens (Human).
Check to include this hit in error tolerant search or archive report

Query	Observed	Mr(expt)	Mr(calc)	Delta	Miss	Score	Expect	Rank	Peptide
196	665.3678	1328.7210	1328.7187	0.0023	0	62	0.0032	1	R.NLDLDSIIAEVK.A
260	738.3990	1474.7834	1474.7780	0.0055	0	41	0.33	1	R.FLEQQNQVLQTK.W

Proteins matching the same set of peptides:

A44861 **Mass:** 66110 **Score:** 102 **Queries matched:** 2

keratin, 67K type II epidermal - human

---

8. Q6IG03_RAT **Mass:** 60977 **Score:** 76 **Queries matched:** 2
Type II keratin Kb36.- Rattus norvegicus (Rat).
Check to include this hit in error tolerant search or archive report

Query	Observed	Mr(expt)	Mr(calc)	Delta	Miss	Score	Expect	Rank	Peptide
210	679.3525	1356.6904	1356.7249	-0.0344	0	35	1.4	3	R.NLDLDSIIAEVR.A
260	738.3990	1474.7834	1474.7780	0.0055	0	41	0.33	1	R.FLEQQNQVLQTK.W

Proteins matching the same set of peptides:
Q6NXH9_MOUSE **Mass:** 59502 **Score:** 76 **Queries matched:** 2
Type II keratin Kb36.- Mus musculus (Mouse).

---

9. Q77GM4_LSDV **Mass:** 29074 **Score:** 76 **Queries matched:** 2
Putative DNA-binding virion core protein.- Lumpy skin disease virus NW-LW.
Check to include this hit in error tolerant search or archive report

Query	Observed	Mr(expt)	Mr(calc)	Delta	Miss	Score	Expect	Rank	Peptide
183	**653.8817**	**1305.7489**	**1305.7445**	**0.0044**	**0**	**45**	**0.16**	**1**	**R.SFLSIFNIIPR.N**
406	**606.3156**	**1815.9250**	**1815.9002**	**0.0248**	**0**	**31**	**2.8**	**1**	**K.EIDEYSNKPLQEPVR.L**

---

10. KRBOVI **Mass:** 54986 **Score:** 66 **Queries matched:** 2
keratin, 54K type I cytoskeletal - bovine
Check to include this hit in error tolerant search or archive report

Query	Observed	Mr(expt)	Mr(calc)	Delta	Miss	Score	Expect	Rank	Peptide
211	**679.3672**	**1356.7198**	**1356.7109**	**0.0089**	**1**	**12**	**2.9e+02**	**3**	**R.QSVEADINGLRR.V**
224	**695.8337**	**1389.6528**	**1389.6735**	**-0.0208**	**0**	**54**	**0.021**	**1**	**K.QSLEASLAETEGR.Y**

Proteins matching the same set of peptides:
KRHU0 **Mass:** 59720 **Score:** 66 **Queries matched:** 2
keratin 10, type I, cytoskeletal - human
K1C10_HUMAN **Mass:** 59711 **Score:** 66 **Queries matched:** 2
Keratin, type I cytoskeletal 10 (Cytokeratin-10) (CK-10) (Keratin-10) (K10).- Homo sapiens (Human).
KRMSE1 **Mass:** 57847 **Score:** 66 **Queries matched:** 2
keratin, 59K type I cytoskeletal - mouse
Q2ENC7_RABIT **Mass:** 31541 **Score:** 66 **Queries matched:** 2
Keratin 10 (Fragment).- Oryctolagus cuniculus (Rabbit).
Q6IFW6_RAT **Mass:** 56699 **Score:** 66 **Queries matched:** 2
Type I keratin KA10.- Rattus norvegicus (Rat).
Q8BUX3_MOUSE **Mass:** 49642 **Score:** 66 **Queries matched:** 2
16 days embryo head cDNA, RIKEN full-length enriched library, clone:C130087C14 product:EPIDERMAL KE
Q8BV09_MOUSE **Mass:** 52824 **Score:** 66 **Queries matched:** 2
16 days embryo head cDNA, RIKEN full-length enriched library, clone:C130014E24 product:KERATIN, TYP
Q8BVU3_MOUSE **Mass:** 57178 **Score:** 66 **Queries matched:** 2
0 day neonate head cDNA, RIKEN full-length enriched library, clone:4833432L21 product:KERATIN, TYPE
Q8N175_HUMAN **Mass:** 59020 **Score:** 66 **Queries matched:** 2
Keratin 10.- Homo sapiens (Human).
Q9CXH6_MOUSE **Mass:** 58758 **Score:** 66 **Queries matched:** 2
17 days embryo head cDNA, RIKEN full-length enriched library, clone:3300001N19 product:inferred: pu
A31994 **Mass:** 57384 **Score:** 66 **Queries matched:** 2
keratin 10, type I, epidermal - human
AAA39391 **Mass:** 57978 **Score:** 66 **Queries matched:** 2
MUSKTEPI2 NID: - Mus musculus
AAA59199 **Mass:** 39832 **Score:** 66 **Queries matched:** 2
HUMK10A NID: - Homo sapiens
AAA59468 **Mass:** 46473 **Score:** 66 **Queries matched:** 2
HUMKRT10A NID: - Homo sapiens

---

11. Q9DHL2_YLDV **Mass:** 103662 **Score:** 64 **Queries matched:** 4
101L protein.- Yaba-like disease virus (YLDV).
Check to include this hit in error tolerant search or archive report

Query	Observed	Mr(expt)	Mr(calc)	Delta	Miss	Score	Expect	Rank	Peptide
75	531.7860	1061.5574	1061.5505	0.0069	0	(28)	8.4	1	R.DFENLLGVR.S
76	531.7903	1061.5660	1061.5505	0.0155	0	28	7.9	1	R.DFENLLGVR.S
77	531.7946	1061.5747	1061.5505	0.0242	0	(18)	97	1	R.DFENLLGVR.S
131	403.5504	1207.6293	1207.6197	0.0097	0	36	1.1	2	K.LNDDGSPHLIK.K

---

12. Q6PVZ3_CHICK **Mass:** 57268 **Score:** 62 **Queries matched:** 1
Type II alpha-keratin IIC.- Gallus gallus (Chicken).
Check to include this hit in error tolerant search or archive report

Query	Observed	Mr(expt)	Mr(calc)	Delta	Miss	Score	Expect	Rank	Peptide
196	665.3678	1328.7210	1328.7187	0.0023	0	62	0.0032	1	R.NLDLDSIIADLK.A

13. Q90ZF7_RANCA **Score:** 62 **Queries matched:** 1
Keratin 8.- Rana catesbeiana (Bull frog).
☐ Check to include this hit in error tolerant search or archive report

Query	Observed	Mr(expt)	Mr(calc)	Delta	Miss	Score	Expect	Rank	Peptide
196	665.3678	1328.7210	1328.7187	0.0023	0	62	0.0032	1	R.NLDLDSILAEVK.A

14. K2C8_MOUSE **Mass:** 54400 **Score:** 56 **Queries matched:** 1
Keratin, type II cytoskeletal 8 (Cytokeratin-8) (CK-8) (Keratin-8) (K8) (Cytokeratin endo A).- Mus
☐ Check to include this hit in error tolerant search or archive report

Query	Observed	Mr(expt)	Mr(calc)	Delta	Miss	Score	Expect	Rank	Peptide
222	692.3469	1382.6792	1382.7194	-0.0402	1	56	0.01	1	K.SLNNKFASFIDK.V

Proteins matching the same set of peptides:
JT0407 **Mass:** 54277 **Score:** 56 **Queries matched:** 1
keratin 8, type II cytoskeletal, embryonic - mouse
JS0658 **Mass:** 54531 **Score:** 56 **Queries matched:** 1
cytokeratin EndoA - mouse
S05474 **Mass:** 54415 **Score:** 56 **Queries matched:** 1
keratin 8, type II, cytoskeletal - mouse
AAI06155 **Mass:** 54514 **Score:** 56 **Queries matched:** 1
BC106154 NID: - Mus musculus
AAA37551 **Mass:** 53210 **Score:** 56 **Queries matched:** 1
MUSENDOAA NID: - Mus musculus
BAE38980 **Mass:** 54546 **Score:** 56 **Queries matched:** 1
AK166737 NID: - Mus musculus
BAE40567 **Mass:** 54459 **Score:** 56 **Queries matched:** 1
AK168726 NID: - Mus musculus

15. T08212 **Score:** 54 **Queries matched:** 3
RNA-directed RNA polymerase (EC 2.7.7.48) - Hendra virus
☐ Check to include this hit in error tolerant search or archive report

Query	Observed	Mr(expt)	Mr(calc)	Delta	Miss	Score	Expect	Rank	Peptide
36	421.7629	841.5113	841.4810	0.0303	0	33	2.8	7	R.LGTWLPR.G
132	**403.8745**	**1208.6018**	**1208.5673**	**0.0345**	**0**	**14**	**1.7e+02**	**10**	**R.VPYVGSSTDER.S**
439	663.3539	1987.0398	1986.9356	0.1042	1	7	8.8e+02	9	K.SFEEDLELATFLMDRR.I + Oxidation (M)

Proteins matching the same set of peptides:
AAC83194 **Score:** 54 **Queries matched:** 3

16. Q7RG06_PLAYO **Mass:** 128859 **Score:** 48 **Queries matched:** 2
Mitochondrial carrier protein, putative.- Plasmodium yoelii yoelii.
☐ Check to include this hit in error tolerant search or archive report

	Query	Observed	Mr(expt)	Mr(calc)	Delta	Miss	Score	Expect	Rank	Peptide
	131	403.5504	1207.6293	1207.7441	-0.1147	1	22	26	3	K.KVNLFPHIIK.N
☑	133	**605.3136**	**1208.6127**	**1208.6917**	**-0.0790**	**0**	**27**	**8.2**	**1**	**K.EVNLFPHIIK.N**

17. Q6BL61_DEBHA **Score:** 46 **Queries matched:** 1
Similar to CA4877|IPF1667 Candida albicans IPF1667.- Debaryomyces hansenii (Yeast) (Torulaspora hansenii).
☐ Check to include this hit in error tolerant search or archive report

Query	Observed	Mr(expt)	Mr(calc)	Delta	Miss	Score	Expect	Rank	Peptide
210	679.3525	1356.6904	1356.7864	-0.0959	1	46	0.1	2	K.KTLLEEGIVDIK.D

18. Q2ADE5_9FIRM **Mass:** 54626 **Score:** 44 **Queries matched:** 1
Outer membrane efflux protein precursor.- Halothermothrix orenii H 168.
☐ Check to include this hit in error tolerant search or archive report

	Query	Observed	Mr(expt)	Mr(calc)	Delta	Miss	Score	Expect	Rank	Peptide
☑	113	585.2987	1168.5828	1168.5798	0.0030	0	44	0.24	1	K.QLYVMSGLDK.S + Oxidation (M)

19. Q1UAH7_LACRE **Mass:** 28733 **Score:** 44 **Queries matched:** 3
Peptidase S14, ClpP.- Lactobacillus reuteri 100-23.
☐ Check to include this hit in error tolerant search or archive report

Query	Observed	Mr(expt)	Mr(calc)	Delta	Miss	Score	Expect	Rank	Peptide
44	438.2405	874.4664	874.4396	0.0268	1	44	0.29	1	K.AGDKLDEK.T
45	438.2424	874.4703	874.4396	0.0307	1	(41)	0.53	1	K.AGDKLDEK.T
46	438.2522	874.4899	874.4396	0.0503	1	(29)	8.7	1	K.AGDKLDEK.T

20. Q24EG4_TETTH **Score:** 44 **Queries matched:** 3
Hypothetical protein.- Tetrahymena thermophila SB210.
Check to include this hit in error tolerant search or archive report

Query	Observed	Mr(expt)	Mr(calc)	Delta	Miss	Score	Expect	Rank	Peptide
44	438.2405	874.4664	874.4396	0.0268	1	44	0.29	1	K.QDKIDEK.N
45	438.2424	874.4703	874.4396	0.0307	1	(41)	0.53	1	K.QDKIDEK.N
46	438.2522	874.4899	874.4396	0.0503	1	(29)	8.7	1	K.QDKIDEK.N

Peptide matches not assigned to protein hits: (no details means no match)

Query	Observed	Mr(expt)	Mr(calc)	Delta	Miss	Score	Expect	Rank	Peptide
55	478.7857	955.5568	955.4909	0.0658	0	43	0.27	1	MELLAHAR + Oxidation (M)
166	635.8416	1269.6687	1269.7292	-0.0605	0	42	0.27	1	QLIQSLQEIAK
318	793.8898	1585.7651	1585.7583	0.0068	0	40	0.46	1	VQALEEANNDLENK
64	516.7633	1031.5121	1031.6491	-0.1370	1	38	0.83	1	ILNLYKLR
96	564.7929	1127.5712	1127.6550	-0.0837	1	38	0.85	1	KIEDLGNLVK
28	412.2569	822.4992	822.4276	0.0716	0	38	0.8	1	FFKPPST
40	428.7728	855.5310	855.4814	0.0496	0	38	0.85	1	IATGELPR
22	412.2378	822.4611	822.4276	0.0335	0	37	0.93	1	FFKPPST
32	414.7536	827.4926	827.4977	-0.0051	1	37	1.2	1	INNIAKR
43	438.2365	874.4585	874.4508	0.0077	1	36	1.5	1	ENKIDTR
21	412.2378	822.4611	822.4276	0.0335	0	33	2.2	1	FFKPPST
24	412.2454	822.4763	822.4276	0.0488	0	33	2.5	1	FFKPPST
90	552.7911	1103.5676	1103.5094	0.0582	0	33	3	1	EAVEQNESAK
27	412.2493	822.4840	822.4276	0.0564	0	33	2.6	1	FFKPPST
271	495.9762	1484.9069	1484.8463	0.0606	1	32	1.8	1	QFGVTLPEGIRLR
34	415.2428	828.4710	828.4341	0.0368	0	32	3.8	1	IDIPDTR
30	412.7636	823.5127	823.4916	0.0211	0	32	2.6	1	AALPSIPR
234	709.8809	1417.7472	1417.6507	0.0965	0	31	3.1	1	TAMQDVEAVDSPR
19	412.2321	822.4497	822.4276	0.0221	0	31	3.6	1	FFKPPST
26	412.2493	822.4840	822.4276	0.0564	0	30	4.5	1	FFKPPST
25	412.2454	822.4763	822.4276	0.0488	0	30	4.7	1	FFKPPST
23	412.2435	822.4725	822.4276	0.0450	0	30	4.8	1	FFKPPST
190	658.8825	1315.7505	1315.7096	0.0409	1	30	4.8	1	TIQTDLINKDR
239	723.4124	1444.8102	1444.7674	0.0428	0	30	4.5	1	TVPEVLLSGHHEK
48	460.2246	918.4346	918.5650	-0.1304	0	30	7	1	LGGIPPPIR
237	716.3758	1430.7371	1430.7517	-0.0146	1	29	5	1	AEDASSWRIVLGK
454	1042.0143	2082.0141	2082.0455	-0.0314	1	29	3.4	1	VMNKFVDIELVDGIGDFR + Oxidation (M)
20	412.2321	822.4497	822.4276	0.0221	0	29	5.8	1	FFKPPST
74	531.2951	1060.5756	1060.4019	0.1737	0	29	6.6	1	DTSSICYSE
172	639.8427	1277.6709	1277.7455	-0.0746	1	29	6.7	1	RLDIHDILGVK
98	566.3053	1130.5960	1130.6295	-0.0335	1	28	8.2	1	LLSAKDSLER
297	766.4267	1530.8388	1530.7777	0.0611	0	27	9.9	1	VDLTPEESISQVSK
58	487.7262	973.4378	973.5960	-0.1582	0	27	13	1	KPYLNVIK
358	822.4415	1642.8685	1642.7872	0.0813	0	27	8	1	AEELGIPVLDEDGMR
332	800.4204	1598.8263	1598.8701	-0.0438	1	26	9.9	1	LHSMIVDLDSVVKK + Oxidation (M)
7	386.2337	770.4528	770.4650	-0.0123	0	26	13	1	VSSILPR
108	577.2909	1152.5673	1152.6502	-0.0830	0	26	11	1	IGLSEGHISIK
82	535.3262	1068.6379	1068.5677	0.0701	0	26	11	1	LFEIICFK
368	558.6351	1672.8834	1672.8018	0.0817	1	26	11	1	QFIDKLTEMYEEK
157	622.7733	1243.5320	1243.5067	0.0253	0	25	13	1	MSPDDFEFEK
9	387.2242	772.4338	772.4079	0.0259	0	25	21	1	SPEVSVR
149	612.3076	1222.6007	1222.6339	-0.0332	1	25	14	1	QSLSTSVKAMR + Oxidation (M)
315	787.4491	1572.8837	1572.7316	0.1521	0	25	11	1	EFMCLESLPIYR + Oxidation (M)
125	596.7892	1191.5639	1191.5917	-0.0279	0	25	16	1	EASQAMTSLVR
298	766.9227	1531.8309	1531.7704	0.0605	0	25	15	1	ANQEVLEWLVGMK + Oxidation (M)
221	686.8762	1371.7379	1371.6670	0.0709	0	25	16	1	TANEYINSLGYK
175	645.8023	1289.5900	1289.5888	0.0013	0	24	18	1	ATTSAYSDAFTR
158	623.3375	1244.6605	1244.7856	-0.1251	1	24	20	1	IVILKGQVTFK
6	385.2297	768.4448	768.3701	0.0747	0	24	15	1	MVGPGHR + Oxidation (M)
343	812.3504	1622.6863	1622.8337	-0.1474	0	24	14	1	GFTLNIMIAGESGLGK + Oxidation (M)
81	534.2590	1066.5034	1066.4791	0.0243	1	24	20	1	EYRDANSGR
204	669.8780	1337.7415	1337.8394	-0.0979	1	24	22	1	RIIAILQDIVGK
171	638.8561	1275.6976	1275.6346	0.0630	0	24	21	1	DIAFLPAGTDEK
