# Supplementary material for: Fit-for-purpose curated database application in mass spectrometry-based targeted protein identification and validation
Source: BMC Res Notes. 2014 Jul 10;7:444. doi: 10.1186/1756-0500-7-444 (PMC4102332; doi:10.1186/1756-0500-7-444)
Supplement: Additional file 3 — Sheeppox Virus-band A-PBRdb search. [file 1756-0500-7-444-S3.pdf]

Mascot Search Results

Search title	:	L:\Client Info\Kitching\Data\20061017-PK-GP\20061101-02 20061017-PK-GP band A.wiff (sample number 1)
MS data file	:	L:\Client Info\Kitching\Data\20061017-PK-GP\20061101-02 20061017-PK-GP band A.mgf
Database	:	PBR (21000 sequences; 5863138 residues)
Timestamp	:	22 Nov 2006 at 13:54:13 GMT
Significant hits:	SPPV-A_084	097[Sheeppox virus strain A] ||VBRC_genome_id|1511|VBRC_gene_id|44607|: ; SPPV-A_084 084
	LSDV-WARM_103	101[Lumpy skin disease virus strain Neethling Warmbaths LW] AAN02669.1|22595636|VBRC_genome_id|1497
	SPPV-A_078	090[Sheeppox virus strain A] ||VBRC_genome_id|1511|VBRC_gene_id|44600|: ; SPPV-A_078 078
	DPV-W848_83-108	110[Deerpox virus strain W-848-83] ||VBRC_genome_id|1515|VBRC_gene_id|45221|:
	SPPV-A_068	080[Sheeppox virus strain A] ||VBRC_genome_id|1511|VBRC_gene_id|44590|: ; SPPV-A_068 068
	YMTV-YLD_101	101L[Yaba-like Disease Virus strain Unknown] NP_073486.1|12085084|VBRC_genome_id|1487|VBRC_gene_id|
	GTPV-G20LKV_046	059[Goatpox virus strain G20-LKV] ||VBRC_genome_id|1514|VBRC_gene_id|45021|: ; GTPV-G20LKV_046 046
	DPV-W848_83-091	093[Deerpox virus strain W-848-83] ||VBRC_genome_id|1515|VBRC_gene_id|45204|:
	SFV-KAS_103	s099L[Rabbit fibroma virus strain Kasza] AAF17982.1|6578628|VBRC_genome_id|1480|VBRC_gene_id|38699|
	AMEV-EPB_169	156[Amsacta moorei entomopoxvirus strain Moyer] NP_064938.1|9964470|VBRC_genome_id|1475|VBRC_gene_i
	CNPV-WC93_150	150[Canarypox virus strain ATCC VR111] NP_955173.1|40556088|VBRC_genome_id|1506|VBRC_gene_id|43623|: CNPV

Probability Based Mowse Score

Ions score is -10*Log(P), where P is the probability that the observed match is a random event.
Individual ions scores > 26 indicate identity or extensive homology (p<0.05).
Protein scores are derived from ions scores as a non-probabilistic basis for ranking protein hits.

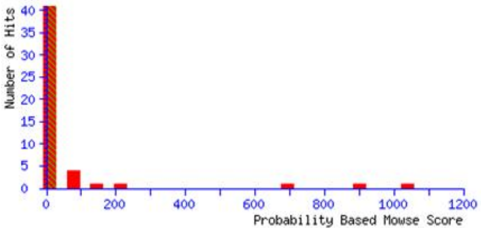

Peptide Summary Report

Format As | Peptide Summary | Help

Significance threshold p< 0.05 | Max. number of hits 20

Standard scoring ● MudPIT scoring ○ | Ions score cut-off 0 | Show sub-sets ☐

Show pop-ups ● Suppress pop-ups ○ | Sort unassigned Decreasing Score | Require bold red ☐

Select All | Select None | Search Selected | ☐ **Error tolerant** | Archive Report

1. SPPV-A_084 **Mass:** 104657 **Score:** 1039 **Queries matched:** 80
097[Sheeppox virus strain A] ||VBRC_genome_id|1511|VBRC_gene_id|44607|: ; SPPV-A_084 084
☐ Check to include this hit in error tolerant search or archive report

	Query	Observed	Mr(expt)	Mr(calc)	Delta	Miss	Score	Expect	Rank	Peptide
☑	8	387.2223	772.4301	772.4330	-0.0029	0	(17)	0.4	1	K.SPELSIK.E
☑	9	387.2242	772.4338	772.4330	0.0008	0	24	0.086	1	K.SPELSIK.E
☑	10	387.2279	772.4412	772.4330	0.0082	0	(19)	0.24	1	K.SPELSIK.E
☑	16	405.2216	808.4286	808.4079	0.0207	0	10	2.4	1	K.YVDISGR.D
☑	19	412.2321	822.4497	822.4752	-0.0255	0	(20)	0.39	1	R.FFQLLR.L
☑	20	412.2321	822.4497	822.4752	-0.0255	0	(16)	0.96	1	R.FFQLLR.L
☑	21	412.2378	822.4611	822.4752	-0.0141	0	22	0.23	1	R.FFQLLR.L
☑	22	412.2378	822.4611	822.4752	-0.0141	0	(18)	0.62	1	R.FFQLLR.L
☑	23	412.2435	822.4725	822.4752	-0.0026	0	(16)	0.89	1	R.FFQLLR.L
☑	24	412.2454	822.4763	822.4752	0.0012	0	(18)	0.64	1	R.FFQLLR.L
☑	25	412.2454	822.4763	822.4752	0.0012	0	(16)	0.87	1	R.FFQLLR.L
☑	26	412.2493	822.4840	822.4752	0.0088	0	(16)	0.84	1	R.FFQLLR.L
☑	27	412.2493	822.4840	822.4752	0.0088	0	(17)	0.67	1	R.FFQLLR.L
☑	28	412.2569	822.4992	822.4752	0.0240	0	(21)	0.28	1	R.FFQLLR.L
☑	29	412.7236	823.4327	823.4262	0.0065	0	27	0.058	1	R.ELLMYR.L
☑	31	414.7536	827.4926	827.4865	0.0062	0	(34)	0.0075	1	R.LNNINIK.Y
☑	32	414.7536	827.4926	827.4865	0.0062	0	37	0.0046	1	R.LNNINIK.Y
☑	43	438.2365	874.4585	874.4548	0.0036	0	36	0.01	1	R.FQDQIPK.L
☑	44	438.2405	874.4664	874.4548	0.0115	0	(25)	0.12	1	R.FQDQIPK.L
☑	45	438.2424	874.4703	874.4548	0.0154	0	(33)	0.02	1	R.FQDQIPK.L
☑	46	438.2522	874.4899	874.4548	0.0351	0	(17)	0.8	1	R.FQDQIPK.L
☑	75	531.7860	1061.5574	1061.5505	0.0069	0	(28)	0.064	1	R.DFENLLGVR.R
☑	76	531.7903	1061.5660	1061.5505	0.0155	0	28	0.06	1	R.DFENLLGVR.R
☑	77	531.7946	1061.5747	1061.5505	0.0242	0	(18)	0.74	1	R.DFENLLGVR.R
☑	78	531.8000	1061.5854	1061.5505	0.0349	0	(4)	16	1	R.DFENLLGVR.R
☑	99	568.8259	1135.6372	1135.6311	0.0062	0	(40)	0.0035	1	K.EVLMLYAGLK.I
☑	100	568.8304	1135.6462	1135.6311	0.0151	0	(11)	2.7	1	K.EVLMLYAGLK.I

	Query	Observed	Mr(expt)	Mr(calc)	Delta	Miss	Score	Expect	Rank	Peptide
☑	104	576.8200	1151.6254	1151.6260	-0.0006	0	44	0.0008	1	K.EVLMLYAGLK.I + Oxidation (M)
☑	110	581.3207	1160.6268	1160.6012	0.0257	0	11	3.2	1	K.QYIHNMLVK.A + Oxidation (M)
☑	113	585.2987	1168.5828	1168.5876	-0.0048	1	(29)	0.03	1	R.LKYPEYNSR.F
☑	114	585.3055	1168.5964	1168.5876	0.0088	1	40	0.0029	1	R.LKYPEYNSR.F
☑	131	403.5504	1207.6293	1207.6197	0.0097	0	41	0.0023	1	K.IDDNGSPHLIK.K
☑	140	611.8180	1221.6214	1221.6353	-0.0139	0	(33)	0.011	1	K.DVLSTSIQGFR.V
☑	141	611.8226	1221.6307	1221.6353	-0.0046	0	(21)	0.16	1	K.DVLSTSIQGFR.V
☑	142	611.8250	1221.6354	1221.6353	0.0000	0	37	0.004	1	K.DVLSTSIQGFR.V
☑	143	611.8250	1221.6354	1221.6353	0.0000	0	(24)	0.087	1	K.DVLSTSIQGFR.V
☑	144	611.8296	1221.6446	1221.6353	0.0093	0	(13)	1	1	K.DVLSTSIQGFR.V
☑	145	611.8319	1221.6493	1221.6353	0.0140	0	(16)	0.52	1	K.DVLSTSIQGFR.V
☑	146	611.8342	1221.6539	1221.6353	0.0186	0	(25)	0.068	1	K.DVLSTSIQGFR.V
☑	147	611.8389	1221.6632	1221.6353	0.0279	0	(23)	0.094	1	K.DVLSTSIQGFR.V
	153	622.2795	1242.5444	1242.5404	0.0040	0	(0)	19	5	R.SINDFDEEFK.M
☑	154	622.2841	1242.5537	1242.5404	0.0133	0	6	4.9	1	R.SINDFDEEFK.M
☑	167	636.2887	1270.5629	1270.5506	0.0124	0	(18)	0.54	1	R.NYQEYYYTK.N
☑	168	636.2911	1270.5677	1270.5506	0.0171	0	18	0.5	1	R.NYQEYYYTK.N
☑	209	678.8099	1355.6052	1355.6135	-0.0083	0	73	8.8e-07	1	R.AMDTDILTMSMK.H
☑	220	686.8148	1371.6150	1371.6084	0.0066	0	(40)	0.0032	1	R.AMDTDILTMSMK.H + Oxidation (M)
☑	223	694.8146	1387.6147	1387.6033	0.0114	0	(38)	0.0024	1	R.AMDTDILTMSMK.H + 2 Oxidation (M)
☑	294	766.4163	1530.8180	1530.8504	-0.0324	0	69	1.6e-06	1	K.LISEVLESLNSISK.K
☑	295	766.4241	1530.8336	1530.8504	-0.0169	0	(14)	0.6	1	K.LISEVLESLNSISK.K
☑	296	766.4267	1530.8388	1530.8504	-0.0117	0	(23)	0.067	1	K.LISEVLESLNSISK.K
☑	297	766.4267	1530.8388	1530.8504	-0.0117	0	(42)	0.00085	1	K.LISEVLESLNSISK.K
☑	320	795.4419	1588.8693	1588.8613	0.0080	0	70	2.6e-06	1	R.DHYYINLLNLLAK.E
☑	321	530.6315	1588.8728	1588.8613	0.0115	0	(52)	0.00018	1	R.DHYYINLLNLLAK.E
☑	322	530.6315	1588.8728	1588.8613	0.0115	0	(49)	0.00037	1	R.DHYYINLLNLLAK.E
☑	323	530.6380	1588.8923	1588.8613	0.0310	0	(50)	0.00026	1	R.DHYYINLLNLLAK.E
☑	331	799.9269	1597.8393	1597.8351	0.0042	0	58	4.5e-05	1	K.LGINYLLDVYSSNK.L
	334	804.4405	1606.8664	1606.8640	0.0024	0	20	0.31	2	K.VISSILPSLCLDYK.V
☑	395	588.6642	1762.9709	1762.9802	-0.0093	0	43	0.0015	1	K.SIIGSNNHTIISVRPR.V
☑	418	636.3100	1905.9083	1905.9367	-0.0284	0	54	7.4e-05	1	K.MLDAANDHNTTLPPLQR.A
☑	419	636.3337	1905.9793	1905.9367	0.0426	0	(8)	2.8	1	K.MLDAANDHNTTLPPLQR.A
☑	421	641.6543	1921.9411	1921.9316	0.0095	0	(46)	0.00057	1	K.MLDAANDHNTTLPPLQR.A + Oxidation (M)
☑	435	663.3000	1986.8782	1986.9839	-0.1057	0	(13)	0.74	1	K.TSYVHPFDVLLHPDYGK.I
☑	436	663.3273	1986.9601	1986.9839	-0.0238	0	62	9e-06	1	K.TSYVHPFDVLLHPDYGK.I
☑	437	663.3418	1987.0036	1986.9839	0.0197	0	(19)	0.16	1	K.TSYVHPFDVLLHPDYGK.I
☑	438	663.3442	1987.0108	1986.9839	0.0269	0	(16)	0.37	1	K.TSYVHPFDVLLHPDYGK.I
☑	439	663.3539	1987.0398	1986.9839	0.0559	0	(33)	0.0072	1	K.TSYVHPFDVLLHPDYGK.I
☑	440	663.3539	1987.0398	1986.9839	0.0559	0	(1)	12	1	K.TSYVHPFDVLLHPDYGK.I
☑	461	713.0257	2136.0553	2136.1367	-0.0814	0	(26)	0.042	1	K.LFHNVINPGYITPNDVVPK.F
☑	462	713.0432	2136.1079	2136.1367	-0.0288	0	(7)	3.3	1	K.LFHNVINPGYITPNDVVPK.F
☑	463	713.0508	2136.1305	2136.1367	-0.0062	0	51	0.00012	1	K.LFHNVINPGYITPNDVVPK.F
☑	464	713.0533	2136.1380	2136.1367	0.0013	0	(33)	0.0079	1	K.LFHNVINPGYITPNDVVPK.F
☑	465	713.0558	2136.1455	2136.1367	0.0088	0	(22)	0.082	1	K.LFHNVINPGYITPNDVVPK.F
☑	466	713.0583	2136.1530	2136.1367	0.0163	0	(36)	0.0039	1	K.LFHNVINPGYITPNDVVPK.F
☑	475	720.0307	2157.0704	2157.0564	0.0140	0	34	0.011	1	K.LISASYDLLHFMVSAGDYR.N
☑	481	733.0410	2196.1011	2196.1102	-0.0091	0	48	0.00071	1	R.FVGGYVKPVEGGFDIQVEEK.M
☑	493	746.7161	2237.1264	2237.1190	0.0074	0	(38)	0.0023	1	K.MHPDLNFENTYLLNLLYK.D
☑	498	752.0466	2253.1179	2253.1139	0.0040	0	43	0.00076	1	K.MHPDLNFENTYLLNLLYK.D + Oxidation (M)
☑	525	1229.6131	2457.2116	2457.1807	0.0309	0	36	0.0033	1	-.MMPINAVTTLDQLEDSEYLFK.V
☑	529	1237.6021	2473.1896	2473.1756	0.0139	0	(21)	0.084	1	-.MMPINAVTTLDQLEDSEYLFK.V + Oxidation (M)
☑	530	1245.5970	2489.1795	2489.1705	0.0089	0	(29)	0.015	1	-.MMPINAVTTLDQLEDSEYLFK.V + 2 Oxidation (M)

Proteins matching the same set of peptides:
SPPV-TU_097 **Mass:** 104657 **Score:** 1039 **Queries matched:** 80
097[Sheeppox virus strain TU-V02127] NP_659673.1|21492554|VBRC_genome_id|1495|VBRC_gene_id|41646|:

---

2. LSDV-WARM_103 **Mass:** 104665 **Score:** 894 **Queries matched:** 70
101[Lumpy skin disease virus strain Neethling Warmbaths LW] AAN02669.1|22595636|VBRC_genome_id|1497

☐ Check to include this hit in error tolerant search or archive report

Query	Observed	Mr(expt)	Mr(calc)	Delta	Miss	Score	Expect	Rank	Peptide
8	387.2223	772.4301	772.4330	-0.0029	0	(17)	0.4	1	K.SPELSIK.E
9	387.2242	772.4338	772.4330	0.0008	0	24	0.086	1	K.SPELSIK.E
10	387.2279	772.4412	772.4330	0.0082	0	(19)	0.24	1	K.SPELSIK.E
16	405.2216	808.4286	808.4079	0.0207	0	10	2.4	1	K.YVDISGR.D
19	412.2321	822.4497	822.4752	-0.0255	0	(20)	0.39	1	R.FFQLLR.L
20	412.2321	822.4497	822.4752	-0.0255	0	(16)	0.96	1	R.FFQLLR.L
21	412.2378	822.4611	822.4752	-0.0141	0	22	0.23	1	R.FFQLLR.L
22	412.2378	822.4611	822.4752	-0.0141	0	(18)	0.62	1	R.FFQLLR.L
23	412.2435	822.4725	822.4752	-0.0026	0	(16)	0.89	1	R.FFQLLR.L
24	412.2454	822.4763	822.4752	0.0012	0	(18)	0.64	1	R.FFQLLR.L
25	412.2454	822.4763	822.4752	0.0012	0	(16)	0.87	1	R.FFQLLR.L
26	412.2493	822.4840	822.4752	0.0088	0	(16)	0.84	1	R.FFQLLR.L
27	412.2493	822.4840	822.4752	0.0088	0	(17)	0.67	1	R.FFQLLR.L
28	412.2569	822.4992	822.4752	0.0240	0	(21)	0.28	1	R.FFQLLR.L
29	412.7236	823.4327	823.4262	0.0065	0	27	0.058	1	R.ELLMYR.L

	31	414.7536	827.4926	827.4865	0.0062	0	(34)	0.0075	1	R.LNNLNIK.Y
	32	414.7536	827.4926	827.4865	0.0062	0	37	0.0046	1	R.LNNLNIK.Y
	43	438.2365	874.4585	874.4548	0.0036	0	36	0.01	1	R.FQDQIPK.L
	44	438.2405	874.4664	874.4548	0.0115	0	(25)	0.12	1	R.FQDQIPK.L
	45	438.2424	874.4703	874.4548	0.0154	0	(33)	0.02	1	R.FQDQIPK.L
	46	438.2522	874.4899	874.4548	0.0351	0	(17)	0.8	1	R.FQDQIPK.L
	75	531.7860	1061.5574	1061.5505	0.0069	0	(28)	0.064	1	R.DFENLLGVR.R
	76	531.7903	1061.5660	1061.5505	0.0155	0	28	0.06	1	R.DFENLLGVR.R
	77	531.7946	1061.5747	1061.5505	0.0242	0	(18)	0.74	1	R.DFENLLGVR.R
	78	531.8000	1061.5854	1061.5505	0.0349	0	(4)	16	1	R.DFENLLGVR.R
	99	568.8259	1135.6372	1135.6311	0.0062	0	(40)	0.0035	1	K.EVLMLYAGLK.I
	100	568.8304	1135.6462	1135.6311	0.0151	0	(11)	2.7	1	K.EVLMLYAGLK.I
	104	576.8200	1151.6254	1151.6260	-0.0006	0	44	0.0008	1	K.EVLMLYAGLK.I + Oxidation (M)
	110	581.3207	1160.6268	1160.6012	0.0257	0	11	3.2	1	K.QYIHNMLVK.A + Oxidation (M)
	113	585.2987	1168.5828	1168.5876	-0.0048	1	(29)	0.03	1	R.LKYPEYNSR.F
	114	585.3055	1168.5964	1168.5876	0.0088	1	40	0.0029	1	R.LKYPEYNSR.F
	131	403.5504	1207.6293	1207.6197	0.0097	0	41	0.0023	1	K.IDDNGSPHLIK.K
	140	611.8180	1221.6214	1221.6353	-0.0139	0	(33)	0.011	1	K.DVLSTSIQGFR.V
	141	611.8226	1221.6307	1221.6353	-0.0046	0	(21)	0.16	1	K.DVLSTSIQGFR.V
	142	611.8250	1221.6354	1221.6353	0.0000	0	37	0.004	1	K.DVLSTSIQGFR.V
	143	611.8250	1221.6354	1221.6353	0.0000	0	(24)	0.087	1	K.DVLSTSIQGFR.V
	144	611.8296	1221.6446	1221.6353	0.0093	0	(13)	1	1	K.DVLSTSIQGFR.V
	145	611.8319	1221.6493	1221.6353	0.0140	0	(16)	0.52	1	K.DVLSTSIQGFR.V
	146	611.8342	1221.6539	1221.6353	0.0186	0	(25)	0.068	1	K.DVLSTSIQGFR.V
	147	611.8389	1221.6632	1221.6353	0.0279	0	(23)	0.094	1	K.DVLSTSIQGFR.V
	167	636.2887	1270.5629	1270.5506	0.0124	0	(18)	0.54	1	R.NYQEYYYTK.N
	168	636.2911	1270.5677	1270.5506	0.0171	0	18	0.5	1	R.NYQEYYYTK.N
	209	678.8099	1355.6052	1355.6135	-0.0083	0	73	8.8e-07	1	R.AMDTDILTMSMK.H
	220	686.8148	1371.6150	1371.6084	0.0066	0	(40)	0.0032	1	R.AMDTDILTMSMK.H + Oxidation (M)
	223	694.8146	1387.6147	1387.6033	0.0114	0	(38)	0.0024	1	R.AMDTDILTMSMK.H + 2 Oxidation (M)
	320	795.4419	1588.8693	1588.8613	0.0080	0	70	2.6e-06	1	R.DHYYINLLNLLAK.E
	321	530.6315	1588.8728	1588.8613	0.0115	0	(52)	0.00018	1	R.DHYYINLLNLLAK.E
	322	530.6315	1588.8728	1588.8613	0.0115	0	(49)	0.00037	1	R.DHYYINLLNLLAK.E
	323	530.6380	1588.8923	1588.8613	0.0310	0	(50)	0.00026	1	R.DHYYINLLNLLAK.E
	331	799.9269	1597.8393	1597.8351	0.0042	0	58	4.5e-05	1	K.LGINYLLDVYSSNK.L
	334	804.4405	1606.8664	1606.8640	0.0024	0	20	0.31	2	K.VISSILPSLCLDYK.V
	395	588.6642	1762.9709	1762.9802	-0.0093	0	43	0.0015	1	K.SIIGSNNHTIISVRPR.V
☑	396	**888.9325**	**1775.8505**	**1775.8406**	**0.0099**	**0**	**27**	**0.066**	**1**	**K.LSLFWDGIDYQEYK.S**
	435	663.3000	1986.8782	1986.9839	-0.1057	0	(13)	0.74	1	K.TSYVHPFDVLLHPDYGK.I
	436	663.3273	1986.9601	1986.9839	-0.0238	0	62	9e-06	1	K.TSYVHPFDVLLHPDYGK.I
	437	663.3418	1987.0036	1986.9839	0.0197	0	(19)	0.16	1	K.TSYVHPFDVLLHPDYGK.I
	438	663.3442	1987.0108	1986.9839	0.0269	0	(16)	0.37	1	K.TSYVHPFDVLLHPDYGK.I
	439	663.3539	1987.0398	1986.9839	0.0559	0	(33)	0.0072	1	K.TSYVHPFDVLLHPDYGK.I
	440	663.3539	1987.0398	1986.9839	0.0559	0	(1)	12	1	K.TSYVHPFDVLLHPDYGK.I
	461	713.0257	2136.0553	2136.1367	-0.0814	0	(26)	0.042	1	K.LFHNVINPGYITPNDVVPK.F
	462	713.0432	2136.1079	2136.1367	-0.0288	0	(7)	3.3	1	K.LFHNVINPGYITPNDVVPK.F
	463	713.0508	2136.1305	2136.1367	-0.0062	0	51	0.00012	1	K.LFHNVINPGYITPNDVVPK.F
	464	713.0533	2136.1380	2136.1367	0.0013	0	(33)	0.0079	1	K.LFHNVINPGYITPNDVVPK.F
	465	713.0558	2136.1455	2136.1367	0.0088	0	(22)	0.082	1	K.LFHNVINPGYITPNDVVPK.F
	466	713.0583	2136.1530	2136.1367	0.0163	0	(36)	0.0039	1	K.LFHNVINPGYITPNDVVPK.F
	475	720.0307	2157.0704	2157.0564	0.0140	0	34	0.011	1	K.LISASYDLLHFMVSAGDYR.N
	481	733.0410	2196.1011	2196.1102	-0.0091	0	48	0.00071	1	R.FVGGYVKPVEGGFDIQVEEK.I
	525	1229.6131	2457.2116	2457.1807	0.0309	0	36	0.0033	1	-.MMPINAVTTLDQLEDSEYLFK.V
	529	1237.6021	2473.1896	2473.1756	0.0139	0	(21)	0.084	1	-.MMPINAVTTLDQLEDSEYLFK.V + Oxidation (M)
	530	1245.5970	2489.1795	2489.1705	0.0089	0	(29)	0.015	1	-.MMPINAVTTLDQLEDSEYLFK.V + 2 Oxidation (M)

Proteins matching the same set of peptides:

LSDV-NEE_149 **Mass:** 104665 **Score:** 894 **Queries matched:** 70
101[Lumpy skin disease virus strain Neethling 2490] NP_150535.1|15150540|VBRC_genome_id|1488|VBRC_g

LSDV-1959_102 **Mass:** 104651 **Score:** 894 **Queries matched:** 70
101[Lumpy skin disease virus strain Neethling vaccine LW 1959] AAN02826.1|22595794|VBRC_genome_id|1

---

3. SPPV-A_078 **Mass:** 76288 **Score:** 700 **Queries matched:** 31
090[Sheeppox virus strain A] ||VBRC_genome_id|1511|VBRC_gene_id|44600|: ; SPPV-A_078 078

☐ Check to include this hit in error tolerant search or archive report

	Query	Observed	Mr(expt)	Mr(calc)	Delta	Miss	Score	Expect	Rank	Peptide
	44	438.2405	874.4664	874.4695	-0.0031	1	(12)	2.4	2	R.QRSLPMK.R + Oxidation (M)
	45	438.2424	874.4703	874.4695	0.0008	1	13	1.8	2	R.QRSLPMK.R + Oxidation (M)
	46	438.2522	874.4899	874.4695	0.0205	1	(5)	13	3	R.QRSLPMK.R + Oxidation (M)
☑	52	**464.2518**	**926.4890**	**926.5007**	**-0.0118**	**0**	**32**	**0.025**	**1**	**R.ANIHTMIK.K**
☑	68	**521.7648**	**1041.5151**	**1041.5130**	**0.0020**	**0**	**52**	**0.00026**	**1**	**K.GEGASFFLSK.Q**
☑	69	**521.7648**	**1041.5151**	**1041.5130**	**0.0020**	**0**	**(9)**	**5.5**	**1**	**K.GEGASFFLSK.Q**
☑	72	**530.8087**	**1059.6029**	**1059.5924**	**0.0105**	**0**	**49**	**0.0004**	**1**	**K.QEILLSTTR.F**
☑	101	**570.7783**	**1139.5420**	**1139.5492**	**-0.0072**	**0**	**32**	**0.028**	**1**	**K.SSLTNSSMSVK.S**
☑	112	**583.7816**	**1165.5486**	**1165.5471**	**0.0015**	**0**	**45**	**0.00073**	**1**	**K.MEEAMSLISR.Q**
☑	119	**591.7884**	**1181.5623**	**1181.5420**	**0.0203**	**0**	**(27)**	**0.054**	**1**	**K.MEEAMSLISR.Q + Oxidation (M)**
☑	187	**656.8641**	**1311.7136**	**1311.7186**	**-0.0051**	**0**	**(44)**	**0.0021**	**1**	**R.NELFELLAHVK.S**
☑	188	**656.8713**	**1311.7280**	**1311.7186**	**0.0094**	**0**	**(38)**	**0.0086**	**1**	**R.NELFELLAHVK.S**
☑	189	**656.8761**	**1311.7376**	**1311.7186**	**0.0190**	**0**	**44**	**0.0021**	**1**	**R.NELFELLAHVK.S**
☑	244	**724.3739**	**1446.7332**	**1446.7289**	**0.0044**	**0**	**51**	**0.00022**	**1**	**K.VNPDNYMLLVNR.L**
☑	245	**724.3890**	**1446.7635**	**1446.7289**	**0.0347**	**0**	**(16)**	**0.66**	**1**	**K.VNPDNYMLLVNR.L**

	Query	Observed	Mr(expt)	Mr(calc)	Delta	Miss	Score	Expect	Rank	Peptide
☑	253	732.3732	1462.7319	1462.7238	0.0081	0	(40)	0.0024	1	K.VNPDNYMLLVNR.L + Oxidation (M)
☑	316	525.9468	1574.8186	1574.8238	-0.0052	1	41	0.0012	1	K.KVNPDNYMLLVNR.L
☑	325	531.2821	1590.8245	1590.8188	0.0057	1	(32)	0.016	1	K.KVNPDNYMLLVNR.L + Oxidation (M)
☑	367	557.3079	1668.9020	1668.8947	0.0073	1	31	0.009	1	R.SNRNELFELLAHVK.S
☑	369	558.6573	1672.9500	1672.9161	0.0338	0	(24)	0.065	1	K.STHPLVLIHSHAHPK.I
☑	370	558.6595	1672.9566	1672.9161	0.0405	0	32	0.01	1	K.STHPLVLIHSHAHPK.I
☑	455	698.3349	2091.9829	2091.9791	0.0037	0	30	0.013	1	K.QQMLMNHITMFDDDLLK.M
☑	456	1051.9661	2101.9177	2101.9228	-0.0052	0	61	1.1e-05	1	K.TSNNNQLDESDFYEWLK.G
☑	457	703.6693	2107.9860	2107.9740	0.0119	0	(18)	0.33	1	K.QQMLMNHITMFDDDLLK.M + Oxidation (M)
☑	459	709.0040	2123.9901	2123.9690	0.0211	0	(24)	0.076	1	K.QQMLMNHITMFDDDLLK.M + 2 Oxidation (M)
☑	491	744.3471	2230.0194	2230.0178	0.0016	1	45	0.0005	1	R.KTSNNNQLDESDFYEWLK.G
☑	500	759.0516	2274.1330	2274.1030	0.0300	0	5	4.2	1	R.DNESTYYIHPIFMSLFGIK.L
☑	513	594.2805	2373.0928	2373.0848	0.0080	0	62	7.8e-06	1	R.FQSIHFVDMSSSSDLAFHYR.D
☑	515	598.2795	2389.0891	2389.0797	0.0094	0	(61)	1.8e-05	1	R.FQSIHFVDMSSSSDLAFHYR.D + Oxidation (M)
☑	518	1209.6478	2417.2811	2417.2689	0.0122	0	54	4.2e-05	1	R.LTEEAPIVFTGISDVISTEIQR.A
☑	526	1232.0778	2462.1411	2462.1449	-0.0037	0	22	0.09	1	K.EDTNTTTVPIDEVASTNDWQVK.L

Proteins matching the same set of peptides:
SPPV-NISKHI_078 **Mass:** 76288 **Score:** 700 **Queries matched:** 31
090[Sheeppox virus strain NISKHI] ||VBRC_genome_id|1512|VBRC_gene_id|44749|: ; SPPV-NISKHI_078 078
SPPV-TU_090 **Mass:** 76288 **Score:** 700 **Queries matched:** 31
090[Sheeppox virus strain TU-V02127] NP_659666.1|21492547|VBRC_genome_id|1495|VBRC_gene_id|41639|:

---

4. DPV-W848_83-108 **Mass:** 105324 **Score:** 207 **Queries matched:** 23
110[Deerpox virus strain W-848-83] ||VBRC_genome_id|1515|VBRC_gene_id|45221|:
☐ Check to include this hit in error tolerant search or archive report

	Query	Observed	Mr(expt)	Mr(calc)	Delta	Miss	Score	Expect	Rank	Peptide
	19	412.2321	822.4497	822.4752	-0.0255	0	(20)	0.39	1	R.FFQLLR.L
	20	412.2321	822.4497	822.4752	-0.0255	0	(16)	0.96	1	R.FFQLLR.L
	21	412.2378	822.4611	822.4752	-0.0141	0	22	0.23	1	R.FFQLLR.L
	22	412.2378	822.4611	822.4752	-0.0141	0	(18)	0.62	1	R.FFQLLR.L
	23	412.2435	822.4725	822.4752	-0.0026	0	(16)	0.89	1	R.FFQLLR.L
	24	412.2454	822.4763	822.4752	0.0012	0	(18)	0.64	1	R.FFQLLR.L
	25	412.2454	822.4763	822.4752	0.0012	0	(16)	0.87	1	R.FFQLLR.L
	26	412.2493	822.4840	822.4752	0.0088	0	(16)	0.84	1	R.FFQLLR.L
	27	412.2493	822.4840	822.4752	0.0088	0	(17)	0.67	1	R.FFQLLR.L
	28	412.2569	822.4992	822.4752	0.0240	0	(21)	0.28	1	R.FFQLLR.L
	43	438.2365	874.4585	874.4548	0.0036	0	36	0.01	1	R.FQDQIPK.L
	44	438.2405	874.4664	874.4548	0.0115	0	(25)	0.12	1	R.FQDQIPK.L
	45	438.2424	874.4703	874.4548	0.0154	0	(33)	0.02	1	R.FQDQIPK.L
	46	438.2522	874.4899	874.4548	0.0351	0	(17)	0.8	1	R.FQDQIPK.L
	131	403.5504	1207.6293	1207.6197	0.0097	0	41	0.0023	1	K.LDDNGSPHLIK.K
☑	339	811.4310	1620.8475	1620.8797	-0.0321	0	(2)	9	1	K.IVSTILPSICLDYK.I
☑	341	811.4444	1620.8742	1620.8797	-0.0054	0	10	1.4	1	K.IVSTILPSICLDYK.I
☑	342	811.4578	1620.9010	1620.8797	0.0213	0	(1)	10	1	K.IVSTILPSICLDYK.I
	395	588.6642	1762.9709	1762.9802	-0.0093	0	35	0.0087	2	K.SIIGSNNHTVITVRPR.I
	396	888.9325	1775.8505	1775.8406	0.0099	0	27	0.066	1	K.LSLFWDGIDYQEYK.S
	525	1229.6131	2457.2116	2457.1807	0.0309	0	36	0.0033	1	-.MMPINAVTTLDQLEDSEYIFK.I
	529	1237.6021	2473.1896	2473.1756	0.0139	0	(21)	0.084	1	-.MMPINAVTTLDQLEDSEYIFK.I + Oxidation (M)
	530	1245.5970	2489.1795	2489.1705	0.0089	0	(29)	0.015	1	-.MMPINAVTTLDQLEDSEYIFK.I + 2 Oxidation (M)

Proteins matching the same set of peptides:
DPV-W1170_84-108 **Mass:** 105319 **Score:** 207 **Queries matched:** 23
110[Deerpox virus strain W-1170-84] ||VBRC_genome_id|1516|VBRC_gene_id|45391|:

---

5. SPPV-A_068 **Mass:** 73433 **Score:** 144 **Queries matched:** 4
080[Sheeppox virus strain A] ||VBRC_genome_id|1511|VBRC_gene_id|44590|: ; SPPV-A_068 068
☐ Check to include this hit in error tolerant search or archive report

	Query	Observed	Mr(expt)	Mr(calc)	Delta	Miss	Score	Expect	Rank	Peptide
	35	415.2447	828.4748	828.5069	-0.0321	1	7	5.4	5	K.NVELVKK.Y
☑	203	669.8513	1337.6881	1337.6867	0.0015	0	61	1.7e-05	1	R.YNDEIITVPFK.L
☑	227	699.9470	1397.8794	1397.8646	0.0148	0	28	0.045	1	K.VYILVPNINILK.I
☑	364	830.4257	1658.8369	1658.8403	-0.0034	0	48	0.00058	1	R.IYSILESISENYTK.E

Proteins matching the same set of peptides:
SPPV-NISKHI_068 **Mass:** 73433 **Score:** 144 **Queries matched:** 4
080[Sheeppox virus strain NISKHI] ||VBRC_genome_id|1512|VBRC_gene_id|44739|: ; SPPV-NISKHI_068 068
SPPV-TU_080 **Mass:** 73419 **Score:** 144 **Queries matched:** 4
080[Sheeppox virus strain TU-V02127] NP_659656.1|21492537|VBRC_genome_id|1495|VBRC_gene_id|41629|:

---

6. YMTV-YLD_101 **Mass:** 103662 **Score:** 97 **Queries matched:** 18
101L[Yaba-like Disease Virus strain Unknown] NP_073486.1|12085084|VBRC_genome_id|1487|VBRC_gene_id|
☐ Check to include this hit in error tolerant search or archive report

Query	Observed	Mr(expt)	Mr(calc)	Delta	Miss	Score	Expect	Rank	Peptide
19	412.2321	822.4497	822.4752	-0.0255	0	(20)	0.39	1	K.FFQLLR.L
20	412.2321	822.4497	822.4752	-0.0255	0	(16)	0.96	1	K.FFQLLR.L

21	412.2378	822.4611	822.4752	-0.0141	0	22	0.23	1	K.FFQLLR.L
22	412.2378	822.4611	822.4752	-0.0141	0	(18)	0.62	1	K.FFQLLR.L
23	412.2435	822.4725	822.4752	-0.0026	0	(16)	0.89	1	K.FFQLLR.L
24	412.2454	822.4763	822.4752	0.0012	0	(18)	0.64	1	K.FFQLLR.L
25	412.2454	822.4763	822.4752	0.0012	0	(16)	0.87	1	K.FFQLLR.L
26	412.2493	822.4840	822.4752	0.0088	0	(16)	0.84	1	K.FFQLLR.L
27	412.2493	822.4840	822.4752	0.0088	0	(17)	0.67	1	K.FFQLLR.L
28	412.2569	822.4992	822.4752	0.0240	0	(21)	0.28	1	K.FFQLLR.L
75	531.7860	1061.5574	1061.5505	0.0069	0	(28)	0.064	1	R.DFENLLGVR.S
76	531.7903	1061.5660	1061.5505	0.0155	0	28	0.06	1	R.DFENLLGVR.S
77	531.7946	1061.5747	1061.5505	0.0242	0	(18)	0.74	1	R.DFENLLGVR.S
78	531.8000	1061.5854	1061.5505	0.0349	0	(4)	16	1	R.DFENLLGVR.S
131	403.5504	1207.6293	1207.6197	0.0097	0	36	0.0067	3	K.LNDDGSPHLIK.K
339	811.4310	1620.8475	1620.8797	-0.0321	0	(2)	9	1	K.VISTILPSLCLDYK.V
341	811.4444	1620.8742	1620.8797	-0.0054	0	10	1.4	1	K.VISTILPSLCLDYK.V
342	811.4578	1620.9010	1620.8797	0.0213	0	(1)	10	1	K.VISTILPSLCLDYK.V

---

7. GTPV-G20LKV_046 **Mass:** 29086 **Score:** 76 **Queries matched:** 2
059[Goatpox virus strain G20-LKV] ||VBRC_genome_id|1514|VBRC_gene_id|45021|: ; GTPV-G20LKV_046 046
☐ Check to include this hit in error tolerant search or archive report

	Query	Observed	Mr(expt)	Mr(calc)	Delta	Miss	Score	Expect	Rank	Peptide
☑	183	**653.8817**	**1305.7489**	**1305.7445**	**0.0044**	**0**	**45**	**0.00091**	**1**	**R.SFLSIFNIIPR.N**
☑	406	**606.3156**	**1815.9250**	**1815.9002**	**0.0248**	**0**	**31**	**0.016**	**1**	**K.EIDEYSNKPLQEPVR.L**

Proteins matching the same set of peptides:
GTPV-Pellor_045 **Mass:** 29086 **Score:** 76 **Queries matched:** 2
059[Goatpox virus strain Pellor] ||VBRC_genome_id|1513|VBRC_gene_id|44870|: ; GTPV-Pellor_045 045
SPPV-A_046 **Mass:** 29087 **Score:** 76 **Queries matched:** 2
059[Sheeppox virus strain A] ||VBRC_genome_id|1511|VBRC_gene_id|44569|: ; SPPV-A_046 046
SPPV-NISKHI_046 **Mass:** 29087 **Score:** 76 **Queries matched:** 2
059[Sheeppox virus strain NISKHI] ||VBRC_genome_id|1512|VBRC_gene_id|44718|: ; SPPV-NISKHI_046 046
SPPV-TU_059 **Mass:** 29087 **Score:** 76 **Queries matched:** 2
059[Sheeppox virus strain TU-V02127] NP_659635.1|21492516|VBRC_genome_id|1495|VBRC_gene_id|41608|:
LSDV-WARM_065 **Mass:** 29074 **Score:** 76 **Queries matched:** 2
063[Lumpy skin disease virus strain Neethling Warmbaths LW] AAN02631.1|22595598|VBRC_genome_id|1497
LSDV-NEE_090 **Mass:** 29074 **Score:** 76 **Queries matched:** 2
063[Lumpy skin disease virus strain Neethling 2490] NP_150497.1|15150502|VBRC_genome_id|1488|VBRC_g
LSDV-1959_064 **Mass:** 29074 **Score:** 76 **Queries matched:** 2
063[Lumpy skin disease virus strain Neethling vaccine LW 1959] AAN02788.1|22595756|VBRC_genome_id|1

---

8. DPV-W848_83-091 **Mass:** 73359 **Score:** 56 **Queries matched:** 2
093[Deerpox virus strain W-848-83] ||VBRC_genome_id|1515|VBRC_gene_id|45204|:
☐ Check to include this hit in error tolerant search or archive report

Query	Observed	Mr(expt)	Mr(calc)	Delta	Miss	Score	Expect	Rank	Peptide
203	669.8513	1337.6881	1337.7231	-0.0349	1	29	0.03	2	R.YKDDIITVPFK.I
227	699.9470	1397.8794	1397.8646	0.0148	0	28	0.045	1	K.VYILVPNINILK.I

Proteins matching the same set of peptides:
DPV-W1170_84-091 **Mass:** 73359 **Score:** 56 **Queries matched:** 2
093[Deerpox virus strain W-1170-84] ||VBRC_genome_id|1516|VBRC_gene_id|45374|:

---

9. SFV-KAS_103 **Mass:** 104195 **Score:** 44 **Queries matched:** 14
s099L[Rabbit fibroma virus strain Kasza] AAF17982.1|6578628|VBRC_genome_id|1480|VBRC_gene_id|38699|
☐ Check to include this hit in error tolerant search or archive report

Query	Observed	Mr(expt)	Mr(calc)	Delta	Miss	Score	Expect	Rank	Peptide
19	412.2321	822.4497	822.4752	-0.0255	0	(20)	0.39	1	R.FFQLLR.L
20	412.2321	822.4497	822.4752	-0.0255	0	(16)	0.96	1	R.FFQLLR.L
21	412.2378	822.4611	822.4752	-0.0141	0	22	0.23	1	R.FFQLLR.L
22	412.2378	822.4611	822.4752	-0.0141	0	(18)	0.62	1	R.FFQLLR.L
23	412.2435	822.4725	822.4752	-0.0026	0	(16)	0.89	1	R.FFQLLR.L
24	412.2454	822.4763	822.4752	0.0012	0	(18)	0.64	1	R.FFQLLR.L
25	412.2454	822.4763	822.4752	0.0012	0	(16)	0.87	1	R.FFQLLR.L
26	412.2493	822.4840	822.4752	0.0088	0	(16)	0.84	1	R.FFQLLR.L
27	412.2493	822.4840	822.4752	0.0088	0	(17)	0.67	1	R.FFQLLR.L
28	412.2569	822.4992	822.4752	0.0240	0	(21)	0.28	1	R.FFQLLR.L
131	403.5504	1207.6293	1207.6561	-0.0267	0	12	1.8	4	K.INDTGTPHLIK.K
339	811.4310	1620.8475	1620.8797	-0.0321	0	(2)	9	1	K.VISTILPSICLDYK.V
341	811.4444	1620.8742	1620.8797	-0.0054	0	10	1.4	1	K.VISTILPSICLDYK.V
342	811.4578	1620.9010	1620.8797	0.0213	0	(1)	10	1	K.VISTILPSICLDYK.V

---

10. AMEV-EPB_169 **Mass:** 147234 **Score:** 37 **Queries matched:** 2
156[Amsacta moorei entomopoxvirus strain Moyer] NP_064938.1|9964470|VBRC_genome_id|1475|VBRC_gene_i
☐ Check to include this hit in error tolerant search or archive report

Query	Observed	Mr(expt)	Mr(calc)	Delta	Miss	Score	Expect	Rank	Peptide
31	414.7536	827.4926	827.4865	0.0062	0	(31)	0.016	3	K.INNIINK.I
32	414.7536	827.4926	827.4865	0.0062	0	37	0.0046	1	K.INNIINK.I

11. CNPV-WC93_150 **Score:** 34 **Queries matched:** 4
150[Canarypox virus strain ATCC VR111] NP_955173.1|40556088|VBRC_genome_id|1506|VBRC_gene_id|43623|: CNPV150 ankyrin repeat protein; (
Check to include this hit in error tolerant search or archive report

	Query	Observed	Mr(expt)	Mr(calc)	Delta	Miss	Score	Expect	Rank	Peptide
	31	414.7536	827.4926	827.4865	0.0062	0	(14)	0.81	4	K.NINIINK.T
	32	414.7536	827.4926	827.4865	0.0062	0	18	0.3	4	K.NINIINK.T
✓	34	**415.2428**	**828.4710**	**828.4705**	**0.0005**	**0**	**15**	**0.72**	**1**	**K.ILDNNLK.H**
	35	415.2447	828.4748	828.4705	0.0043	0	(4)	11	6	K.ILDNNLK.H

12. MPXV-ZRE_121 **Mass:** 103014 **Score:** 22 **Queries matched:** 11
A11L[Monkeypox virus strain Zaire] NP_536548.1|17975034|VBRC_genome_id|1489|VBRC_gene_id|40483|: A1
Check to include this hit in error tolerant search or archive report

	Query	Observed	Mr(expt)	Mr(calc)	Delta	Miss	Score	Expect	Rank	Peptide
	19	412.2321	822.4497	822.4752	-0.0255	0	(20)	0.39	1	R.FFQLLR.L
	20	412.2321	822.4497	822.4752	-0.0255	0	(16)	0.96	1	R.FFQLLR.L
	21	412.2378	822.4611	822.4752	-0.0141	0	22	0.23	1	R.FFQLLR.L
	22	412.2378	822.4611	822.4752	-0.0141	0	(18)	0.62	1	R.FFQLLR.L
	23	412.2435	822.4725	822.4752	-0.0026	0	(16)	0.89	1	R.FFQLLR.L
	24	412.2454	822.4763	822.4752	0.0012	0	(18)	0.64	1	R.FFQLLR.L
	25	412.2454	822.4763	822.4752	0.0012	0	(16)	0.87	1	R.FFQLLR.L
	26	412.2493	822.4840	822.4752	0.0088	0	(16)	0.84	1	R.FFQLLR.L
	27	412.2493	822.4840	822.4752	0.0088	0	(17)	0.67	1	R.FFQLLR.L
	28	412.2569	822.4992	822.4752	0.0240	0	(21)	0.28	1	R.FFQLLR.L
✓	442	**669.3000**	**2004.8782**	**2004.9039**	**-0.0258**	**0**	**0**	**9.8**	**1**	**K.HLIMYEQYFVNDYDR.V**

Proteins matching the same set of peptides:
MPXV-Zaire_1979_005-123 **Mass:** 103014 **Score:** 22 **Queries matched:** 11
126[Monkeypox virus strain Zaire_1979-005] AAY97321.1|68449200|VBRC_genome_id|1523|VBRC_gene_id|469
MPXV-Congo_2003_358-122 **Mass:** 103014 **Score:** 22 **Queries matched:** 11
126[Monkeypox virus strain Congo_2003_358] AAY97120.1|68448998|VBRC_genome_id|1524|VBRC_gene_id|471

13. DPV-W848_83-002 **Mass:** 19489 **Score:** 22 **Queries matched:** 1
002[Deerpox virus strain W-848-83] ||VBRC_genome_id|1515|VBRC_gene_id|45115|:
Check to include this hit in error tolerant search or archive report

	Query	Observed	Mr(expt)	Mr(calc)	Delta	Miss	Score	Expect	Rank	Peptide
✓	6	**385.2297**	**768.4448**	**768.3033**	**0.1414**	**0**	**22**	**0.18**	**1**	**K.MVEMVE.- + 2 Oxidation (M)**

Proteins matching the same set of peptides:
DPV-W848_83-169 **Mass:** 19489 **Score:** 22 **Queries matched:** 1
171[Deerpox virus strain W-848-83] ||VBRC_genome_id|1515|VBRC_gene_id|45282|:
DPV-W1170_84-002 **Mass:** 19781 **Score:** 22 **Queries matched:** 1
002[Deerpox virus strain W-1170-84] ||VBRC_genome_id|1516|VBRC_gene_id|45285|:
DPV-W1170_84-169 **Mass:** 19781 **Score:** 22 **Queries matched:** 1
171[Deerpox virus strain W-1170-84] ||VBRC_genome_id|1516|VBRC_gene_id|45452|:

14. FWPV-FCV_174 **Mass:** 102717 **Score:** 20 **Queries matched:** 1
174[Fowlpox virus strain Iowa] NP_039137.1|7271672|VBRC_genome_id|1476|VBRC_gene_id|37906|: ORF FPV
Check to include this hit in error tolerant search or archive report

	Query	Observed	Mr(expt)	Mr(calc)	Delta	Miss	Score	Expect	Rank	Peptide
✓	334	804.4405	1606.8664	1606.8640	0.0024	0	20	0.29	1	K.LISSVLPSLCLDYK.I

Proteins matching the same set of peptides:
FWPV-HP438_164 **Mass:** 102717 **Score:** 20 **Queries matched:** 1
fp9.174[Fowlpox virus strain HP1-438 Munich] CAE52711.1|41023457|VBRC_genome_id|1509|VBRC_gene_id|4

15. SWPV-NEB_091 **Mass:** 75712 **Score:** 18 **Queries matched:** 1
091[Swinepox virus strain Nebraska 17077-99] NP_570251.1|18640177|VBRC_genome_id|1490|VBRC_gene_id|
Check to include this hit in error tolerant search or archive report

	Query	Observed	Mr(expt)	Mr(calc)	Delta	Miss	Score	Expect	Rank	Peptide
✓	248	**727.8163**	**1453.6181**	**1453.5932**	**0.0249**	**0**	**18**	**0.32**	**1**	**R.NTFSGECYTGYR.S**

16. AMEV-EPB_016 **Mass:** 99476 **Score:** 17 **Queries matched:** 2
003[Amsacta moorei entomopoxvirus strain Moyer] NP_064785.1|9964317|VBRC_genome_id|1475|VBRC_gene_i
Check to include this hit in error tolerant search or archive report

	Query	Observed	Mr(expt)	Mr(calc)	Delta	Miss	Score	Expect	Rank	Peptide
✓	96	**564.7929**	**1127.5712**	**1127.6087**	**-0.0375**	**1**	**8**	**6.8**	**1**	**K.NLHFDNIKK.D**
✓	210	**679.3525**	**1356.6904**	**1356.7612**	**-0.0708**	**0**	**10**	**2**	**1**	**R.NIIDNNTLSIIK.T**

17. MSEV-TUC_257 **Mass:** 84138 **Score:** 15 **Queries matched:** 2

257[Melanoplus sanguinipes entomopoxvirus strain Tucson] NP_048328.1|9631420|VBRC_genome_id|1478|VB

Check to include this hit in error tolerant search or archive report

Query	Observed	Mr(expt)	Mr(calc)	Delta	Miss	Score	Expect	Rank	Peptide
34	415.2428	828.4710	828.4705	0.0005	0	15	0.72	1	K.IIDNLNK.L
35	415.2447	828.4748	828.4705	0.0043	0	(4)	11	6	K.IIDNLNK.L

---

18. AMEV-EPB_183 **Score:** 14 **Queries matched:** 2

170[Amsacta moorei entomopoxvirus strain Moyer] NP_064952.1|9964484|VBRC_genome_id|1475|VBRC_gene_id|37623|: AMV170; DNA-DIRECTED RNA

Check to include this hit in error tolerant search or archive report

Query	Observed	Mr(expt)	Mr(calc)	Delta	Miss	Score	Expect	Rank	Peptide
34	415.2428	828.4710	828.4527	0.0182	0	(8)	4.1	4	K.MPNLINK.L
35	415.2447	828.4748	828.4527	0.0221	0	14	1.1	2	K.MPNLINK.L

---

19. MYXV-LAU_080 **Mass:** 97052 **Score:** 13 **Queries matched:** 1

m076R[Myxoma virus strain Lausanne] NP_051790.1|9633712|VBRC_genome_id|1479|VBRC_gene_id|38505|: m7

Check to include this hit in error tolerant search or archive report

Query	Observed	Mr(expt)	Mr(calc)	Delta	Miss	Score	Expect	Rank	Peptide
125	596.7892	1191.5639	1191.6611	-0.0973	1	13	1.8	1	**K.EHKVDPAAVVK.Y**

---

20. AMEV-EPB_153 **Mass:** 36632 **Score:** 13 **Queries matched:** 2

140[Amsacta moorei entomopoxvirus strain Moyer] NP_064922.1|9964454|VBRC_genome_id|1475|VBRC_gene_i

Check to include this hit in error tolerant search or archive report

Query	Observed	Mr(expt)	Mr(calc)	Delta	Miss	Score	Expect	Rank	Peptide
34	415.2428	828.4710	828.5069	-0.0359	1	(7)	4.9	6	K.LNDLVKK.D
35	415.2447	828.4748	828.5069	-0.0321	1	14	0.94	1	K.LNDLVKK.D

Peptide matches not assigned to protein hits: (no details means no match)

Query	Observed	Mr(expt)	Mr(calc)	Delta	Miss	Score	Expect	Rank	Peptide
85	543.2877	1084.5608	1084.5375	0.0233	1	12	2.3	1	ENIFKCFK
153	622.2795	1242.5444	1242.5406	0.0037	0	12	1.3	1	LMASMATEMSR + Oxidation (M)
273	496.2562	1485.7467	1485.6843	0.0624	1	12	1.5	1	DQLFLSEMVKMN + 2 Oxidation (M)
290	758.9405	1515.8664	1515.8330	0.0334	0	11	2.3	1	VSVTIIPLMADSVR + Oxidation (M)
82	535.3262	1068.6379	1068.6655	-0.0276	1	10	3	1	IINIIKNNK
49	461.2877	920.5608	920.4087	0.1521	0	10	2.2	1	ENISESDK
64	516.7633	1031.5121	1031.6127	-0.1006	1	10	2	1	LINFKELR
291	758.9405	1515.8664	1515.8330	0.0334	0	9	3.4	1	VSVTIIPLMADSVR + Oxidation (M)
196	665.3678	1328.7210	1328.6935	0.0275	1	9	2.9	1	INNNDEELIKK
216	685.3726	1368.7306	1368.7435	-0.0129	0	8	6.1	1	TIMLNGIVDLHK + Oxidation (M)
185	655.8644	1309.7142	1309.6224	0.0919	0	8	5.1	1	DSAIMVASEPYK
89	552.3000	1102.5854	1102.6597	-0.0743	0	7	6.3	1	IGTLLVSSSVK
284	504.2849	1509.8330	1509.7894	0.0435	1	7	5	1	VTSKMLETMLSVR + Oxidation (M)
95	558.8102	1115.6059	1115.6008	0.0051	0	7	6.4	1	MVTPIELASR
285	756.4180	1510.8215	1510.8970	-0.0755	0	7	8.2	1	SILLVNPSSIDLLK
36	421.7629	841.5113	841.5385	-0.0272	1	6	8	1	RDIVIVK
166	635.8416	1269.6687	1269.6717	-0.0030	1	6	3.7	1	LQSLYNKYNK
162	630.8562	1259.6978	1259.6267	0.0711	1	6	7.8	1	WPMLAMREAR
129	599.3537	1196.6929	1196.7128	-0.0199	1	6	11	1	LPDLIKELTR
87	544.7907	1087.5668	1087.4426	0.1242	1	5	9.3	1	MMKDYDNR + Oxidation (M)
336	806.3757	1610.7369	1610.7365	0.0004	0	5	12	1	DHYNTIVDWASYK
207	674.3378	1346.6610	1346.6929	-0.0319	0	4	15	1	TGTLTQIDEIEK
128	599.3216	1196.6286	1196.7856	-0.1570	1	4	15	1	LLIDRITLIK
40	428.7728	855.5310	855.5178	0.0132	0	4	9.6	1	SPSLALLR
257	736.3970	1470.7794	1470.8657	-0.0863	1	4	13	1	IDILSIKLDNISK
134	606.3110	1210.6074	1210.6380	-0.0305	0	4	10	1	LSVMLFTTQR + Oxidation (M)
173	640.3339	1278.6533	1278.6642	-0.0108	0	3	26	1	SSPLELFMLSR
7	386.2337	770.4528	770.4901	-0.0374	0	3	17	1	VALEVLK
365	553.9892	1658.9458	1658.9243	0.0215	1	3	16	1	FSTDKNPSILPSLIK
70	523.2807	1044.5468	1044.5563	-0.0095	1	3	16	1	SKNINLNDK
242	723.8615	1445.7085	1445.7548	-0.0463	1	3	13	1	INEMVDELVTRK
251	731.4039	1460.7932	1460.8139	-0.0207	1	2	17	1	LKFHIVTDAVYR
158	623.3375	1244.6605	1244.7315	-0.0710	0	2	23	1	IIVIPIVCYR
280	501.2501	1500.7284	1500.7502	-0.0218	1	2	12	1	MFRMTVELMSLK + Oxidation (M)
94	558.2405	1114.4665	1114.6233	-0.1568	1	2	18	1	KDEAIEIGLK
337	808.4493	1614.8840	1614.6905	0.1935	0	2	17	1	NLNVDDMMYDEIK + Oxidation (M)
306	779.4443	1556.8741	1556.8093	0.0648	1	2	9.1	1	HSMLTNAISSKVNR
402	897.9125	1793.8104	1793.9532	-0.1428	1	2	8.2	1	TLFTTPMIMRDLVTR
240	723.4124	1444.8102	1444.7561	0.0541	0	2	16	1	SPELVAEVAIFDR
74	531.2951	1060.5756	1060.5036	0.0720	0	2	24	1	NNDITLDEK
234	709.8809	1417.7472	1417.6734	0.0738	0	2	14	1	NICNALYIMYK + Oxidation (M)
239	723.4124	1444.8102	1444.7635	0.0467	1	2	16	1	YKSDIIMVSFVK + Oxidation (M)
