# Supplementary material for: Fit-for-purpose curated database application in mass spectrometry-based targeted protein identification and validation
Source: BMC Res Notes. 2014 Jul 10;7:444. doi: 10.1186/1756-0500-7-444 (PMC4102332; doi:10.1186/1756-0500-7-444)
Supplement: Additional file 4 — Tau-2 NCBInr DB search. [file 1756-0500-7-444-S4.pdf]

MASCOT Search Results

User : Keding
E-mail : chengkeding@gmail.com
Search title : 20120620-test
MS data file : C:\mass_data\Raw data\20130125-01329-prp-s100\05-20130125-Tau-C.RAW
Database : NCBInr 20130202 (22,826,945 sequences; 7,847,231,743 residues)
Timestamp : 4 Feb 2013 at 21:24:22 GMT

Not what you expected? Try **the select summary**.

Search parameters

Score distribution

Legend

Protein Family Summary

Significance threshold p< 0.05 Max. number of families AUTO
Ions score or expect cut-off 0 Dendrograms cut at 0

Preferred taxonomy All entries

Protein families 1–10 (out of 24)

10 per page 1 2 3 Next

1 **gi|2911247** 465 PNS specific microtubule-associated protein tau, ad...

		Score	Mass	Matches	Sequences	emPAI
**1.1**	gi|2911247	465	78830	130 (42)	29 (17)	2.84

PNS specific microtubule-associated protein tau, adult isoform [Homo sapiens]

2 samesets of gi|2911247

130 peptide matches (66 non-duplicate, 64 duplicate)

Query	Dupes	Observed	Mr(expt)	Mr(calc)	ppm	M	Score	Expect	Rank	U	Peptide
5		305.6824	609.3502	609.3486	2.70	0	24	0.47	1	U	K.TPPAPK.T
62		357.7293	713.4440	713.4436	0.69	0	19	0.99	3	U	K.VQIINK.K
65		358.2212	714.4278	714.4276	0.39	0	28	1.4	6	U	K.VQIINK.K + Deamidated (NQ)
71		363.2016	724.3886	724.3868	2.58	0	32	0.37	2	U	R.GAAPPGQK.G
80		378.2243	754.4340	754.4337	0.44	1	25	1.4	5	U	K.KIETHK.L
102		397.2148	792.4150	792.4130	2.59	1	13	1.3	5	U	K.LDFKDR.V
176		431.2380	860.4614	860.4603	1.30	0	30	1.3	2	U	K.IGSTENLK.H
178		431.7297	861.4448	861.4443	0.59	0	47	0.27	2	U	K.IGSTENLK.H + Deamidated (NQ)
182		433.7345	865.4544	865.4545	-0.076	1	38	1.2	2	U	K.SEKLDFK.D
315		490.2636	978.5126	978.5134	-0.78	1	17	1.8	6	U	K.LTFRENAK.A + Deamidated (NQ)
335	3	**498.7531**	**995.4916**	**995.4924**	**-0.73**	**0**	**50**	**0.034**	1	**U**	**K.TPPSSGEPPK.S**
347		**502.2736**	**1002.5326**	**1002.5346**	**-1.91**	**0**	**59**	**0.014**	1	**U**	**K.LDLSNVQSK.C**
454	3	**533.7966**	**1065.5786**	**1065.5819**	**-3.02**	**0**	**48**	**0.015**	1	**U**	**R.TPSLPTPPTR.E**
519	2	**551.2806**	**1100.5466**	**1100.5462**	**0.39**	**0**	**44**	**0.053**	1	**U**	**K.SPVVSGDTSPR.H**
584		566.3204	1130.6262	1130.6295	-2.89	1	53	0.16	1	U	K.KLDLSNVQSK.C
587	2	377.8837	1130.6293	1130.6295	-0.22	1	30	0.57	1	U	K.KLDLSNVQSK.C
591		566.7849	1131.5552	1131.5560	-0.69	0	23	0.42	1	U	K.TDHGAEIVYK.S
592		378.1926	1131.5560	1131.5560	-0.051	0	14	2.4	9	U	K.TDHGAEIVYK.S
596	1	**566.8138**	**1131.6130**	**1131.6135**	**-0.42**	**1**	**58**	**0.046**	1	**U**	**K.KLDLSNVQSK.C + Deamidated (NQ)**
598		378.2122	1131.6148	1131.6135	1.10	1	25	1.2	2	U	K.KLDLSNVQSK.C + Deamidated (NQ)
954	1	**655.3631**	**1308.7116**	**1308.7112**	**0.38**	**0**	**38**	**0.03**	1	**U**	**R.LQTAPVPMPDLK.N**
955		655.3638	1308.7130	1308.7150	-1.49	1	8	1.9	8	U	R.SRTPSLPTPPTR.E
987	5	**663.3600**	**1324.7054**	**1324.7061**	**-0.47**	**0**	**53**	**0.018**	1	**U**	**R.LQTAPVPMPDLK.N + Oxidation (M)**
994	1	**666.3506**	**1330.6866**	**1330.6881**	**-1.09**	**1**	**46**	**0.0091**	1	**U**	**K.AKTDHGAEIVYK.S**
998	1	**444.5701**	**1330.6885**	**1330.6881**	**0.28**	**1**	**59**	**0.0012**	1	**U**	**K.AKTDHGAEIVYK.S**

Query	Dupes	Observed	Mr(expt)	Mr(calc)	ppm	M	Score	Expect	Rank	U	Peptide
1082	2	697.3202	1392.6258	1392.6270	-0.82	0	59	0.00015	1	U	R.SGYSSPGSPGTPGSR.S
1120	1	710.8925	1419.7704	1419.7722	-1.21	1	43	0.17	1	U	R.TPSLPTPPTREPK.K
1122	2	474.2643	1419.7711	1419.7722	-0.77	1	28	0.37	1	U	R.TPSLPTPPTREPK.K
1301	6	526.9448	1577.8126	1577.8162	-2.28	0	36	0.051	1	U	K.IGSLDNITHVPGGGNK.K
1302	2	789.9138	1577.8130	1577.8162	-1.98	0	36	0.062	1	U	K.IGSLDNITHVPGGGNK.K
1309		790.4034	1578.7922	1578.8002	-5.03	0	32	0.21	1	U	K.IGSLDNITHVPGGGNK.K + Deamidated (NQ)
1310		790.4059	1578.7972	1578.8002	-1.86	0	14	1.2	5	U	K.IGSLDNITHVPGGGNK.K + Deamidated (NQ)
1311	1	527.2733	1578.7981	1578.8002	-1.34	0	53	0.0033	1	U	K.IGSLDNITHVPGGGNK.K + Deamidated (NQ)
1421		556.3107	1665.9103	1665.9124	-1.26	1	13	1.4	3	U	R.LQTAPVPMPDLKNVK.S + Oxidation (M)
1480		853.9611	1705.9076	1705.9111	-2.04	1	20	0.61	1	U	K.IGSLDNITHVPGGGNKK.I
1482	1	569.6433	1705.9081	1705.9111	-1.79	1	33	0.024	1	U	K.IGSLDNITHVPGGGNKK.I
1486	3	427.4846	1705.9093	1705.9111	-1.08	1	22	1.3	2	U	K.IGSLDNITHVPGGGNKK.I
1760		652.3244	1953.9514	1953.9531	-0.90	0	34	0.021	1	U	K.STPTAEDVTAPLVDEGAPGK.Q
1763	3	977.9835	1953.9524	1953.9531	-0.35	0	56	0.00043	1	U	K.STPTAEDVTAPLVDEGAPGK.Q
1832	2	495.7783	1979.0841	1979.0840	0.033	0	36	0.021	1	U	K.HVPGGGSVQIVYKPVDLSK.V
1833	4	660.7021	1979.0845	1979.0840	0.22	0	36	0.02	1	U	K.HVPGGGSVQIVYKPVDLSK.V
1908	1	1018.9350	2035.8554	2035.8582	-1.34	0	39	0.0067	1	U	R.QEFEVMEDHAGTYGLGDR.K + Gln->pyro-Glu (N-term Q)
1918		684.9575	2051.8507	2051.8531	-1.19	0	15	0.41	2	U	R.QEFEVMEDHAGTYGLGDR.K + Gln->pyro-Glu (N-term Q); Oxidation (M)
1920	2	1026.9350	2051.8554	2051.8531	1.14	0	65	8.9e-05	1	U	R.QEFEVMEDHAGTYGLGDR.K + Gln->pyro-Glu (N-term Q); Oxidation (M)
1921		1027.4310	2052.8474	2052.8847	-18.2	0	62	0.004	1	U	R.QEFEVMEDHAGTYGLGDR.K
1923		685.3016	2052.8830	2052.8847	-0.85	0	36	0.018	1	U	R.QEFEVMEDHAGTYGLGDR.K
1933	4	690.6324	2068.8754	2068.8796	-2.06	0	50	0.099	1	U	R.QEFEVMEDHAGTYGLGDR.K + Oxidation (M)
1934		1035.4450	2068.8754	2068.8796	-2.03	0	81	8.2e-05	1	U	R.QEFEVMEDHAGTYGLGDR.K + Oxidation (M)
1983		722.3240	2163.9502	2163.9531	-1.37	1	41	0.015	1	U	R.QEFEVMEDHAGTYGLGDRK.D + Gln->pyro-Glu (N-term Q)
1985	2	722.6382	2164.8928	2164.8968	-1.84	0	44	0.0074	1	U	K.DQGGYTMHQDQEGDTDAGLK.E
1986		1083.4540	2164.8934	2164.8968	-1.53	0	48	0.0012	1	U	K.DQGGYTMHQDQEGDTDAGLK.E
1995	1	727.6562	2179.9468	2179.9481	-0.59	1	44	0.00084	1	U	R.QEFEVMEDHAGTYGLGDRK.D + Gln->pyro-Glu (N-term Q); Oxidation (M)
1996		1090.9810	2179.9474	2179.9481	-0.28	1	26	0.81	1	U	R.QEFEVMEDHAGTYGLGDRK.D + Gln->pyro-Glu (N-term Q); Oxidation (M)
1998		1091.4510	2180.8874	2180.8917	-1.94	0	72	2.2e-06	1	U	K.DQGGYTMHQDQEGDTDAGLK.E + Oxidation (M)
2000	3	727.9706	2180.8900	2180.8917	-0.78	0	41	0.0059	1	U	K.DQGGYTMHQDQEGDTDAGLK.E + Oxidation (M)
2002	1	727.9996	2180.9770	2180.9797	-1.24	1	33	0.14	1	U	R.QEFEVMEDHAGTYGLGDRK.D
2003		546.2518	2180.9781	2180.9797	-0.72	1	16	1.1	2	U	R.QEFEVMEDHAGTYGLGDRK.D
2011		550.2499	2196.9705	2196.9746	-1.87	1	33	0.038	1	U	R.QEFEVMEDHAGTYGLGDRK.D + Oxidation (M)
2012	2	733.3309	2196.9709	2196.9746	-1.69	1	36	0.11	1	U	R.QEFEVMEDHAGTYGLGDRK.D + Oxidation (M)
2084		574.2543	2292.9881	2292.9917	-1.58	1	21	0.63	1	U	R.KDQGGYTMHQDQEGDTDAGLK.E
2098		1155.4990	2308.9834	2308.9866	-1.38	1	20	0.18	1	U	R.KDQGGYTMHQDQEGDTDAGLK.E + Oxidation (M)
2099		578.2534	2308.9845	2308.9866	-0.93	1	19	0.96	1	U	R.KDQGGYTMHQDQEGDTDAGLK.E + Oxidation (M)
2100	1	770.6688	2308.9846	2308.9866	-0.89	1	60	0.0001	1	U	R.KDQGGYTMHQDQEGDTDAGLK.E + Oxidation (M)
2126	1	1187.0110	2372.0074	2372.0139	-2.73	0	37	0.016	1	U	K.ESPLQTPTEDGSEEPGSETSDAK.S + Glu->pyro-Glu (N-term E)
2138		797.6801	2390.0185	2390.0245	-2.52	0	33	0.056	1	U	K.ESPLQTPTEDGSEEPGSETSDAK.S
2139		1196.0180	2390.0214	2390.0245	-1.27	0	43	0.00087	1	U	K.ESPLQTPTEDGSEEPGSETSDAK.S

49 subsets and intersections (160 subset proteins in total)

2 — **gi|435476** — 176 — cytokeratin 9 [Homo sapiens]

3

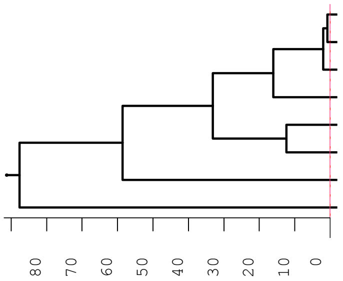

1	**gi|13242237**	154	heat shock cognate 71 kDa protein [Rattus norvegic…
8	**gi|67537918**	57	HS70_TRIRU Heat shock 70 kDa protein [Aspergillu…
6	**gi|144952758**	66	heat shock cognate protein 70 [Scophthalmus maxi…
7	**gi|16507237**	62	78 kDa glucose-regulated protein precursor [Homo …
4	**gi|116511771**	90	molecular chaperone DnaK [Lactococcus lactis subs…
5	**gi|304373393**	68	molecular chaperone DnaK [Mycoplasma hyorhinis …
2	**gi|190613719**	151	Chain B, Crystal Structure Of A Complex Of Sse1p A…
3	**gi|12653415**	131	Heat shock 70kDa protein 9 (mortalin) [Homo sapie…

4

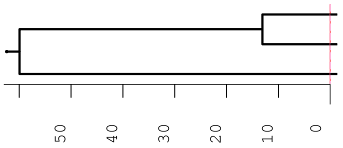

1	**gi|375314771**	152	keratin 1 [Homo sapiens]
2	**gi|297262447**	109	PREDICTED: keratin, type II cytoskeletal 1-like isof…
3	**gi|181402**	92	epidermal cytokeratin 2 [Homo sapiens]

5

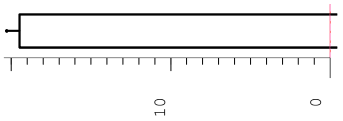

1	**gi|28317**	121	unnamed protein product [Homo sapiens]
2	**gi|119581150**	61	keratin 14 (epidermolysis bullosa simplex, Dowling-…

6 — **gi|3318722** — 118 — Chain E, Leech-Derived Tryptase InhibitorTRYPSIN …

7 — **gi|182087** — 97 — amplaxin [Homo sapiens]

8 — **gi|1827809** — 89 — Chain A, Bovine Mitochondrial F1-Atpase Complexed…

9 — **gi|3065929** — 83 — 14-3-3 protein gamma [Mus musculus]

10 — **gi|12843046** — 76 — unnamed protein product [Mus musculus]

10 per page 1 **2** **3** **Next**

Not what you expected? Try **the select summary**.

Mascot: http://www.matrixscience.com/
