# Supplementary material for: Fit-for-purpose curated database application in mass spectrometry-based targeted protein identification and validation
Source: BMC Res Notes. 2014 Jul 10;7:444. doi: 10.1186/1756-0500-7-444 (PMC4102332; doi:10.1186/1756-0500-7-444)
Supplement: Additional file 5 — Tau-2 custom DB search. [file 1756-0500-7-444-S5.pdf]

MASCOT Search Results

User : Keding
E-mail : chengkeding@gmail.com
Search title : 20120620-test
MS data file : C:\mass_data\Raw data\20130125-01329-prp-s100\05-20130125-Tau-C.RAW
Database : NML_CustomDB 20130307 (18 sequences; 5,836 residues)
Taxonomy : Mammalia (mammals) (18 sequences)
Timestamp : 14 Mar 2013 at 19:38:48 GMT
Warning : No taxonomy indexes for NML_CustomDB, taxonomy 'Mammalia (mammals)' ignored. Searching all entries in NML_CustomDB

Not what you expected? Try **the select summary**.

Search parameters

Score distribution

Legend

Protein Family Summary

Significance threshold p< 0.05 Max. number of families AUTO
Ions score or expect cut-off 0 Dendrograms cut at 0

Preferred taxonomy All entries

Protein families 1–2 (out of 2)

10 per page 1

1 **ni|1000000010|** 1615 Tau-2

		Score	Mass	Matches	Sequences	emPAI
**1.1**	**ni|1000000010|** Tau-2	1615	40273	137 (92)	34 (27)	33.81

137 peptide matches (63 non-duplicate, 74 duplicate)

Query	Dupes	Observed	Mr(expt)	Mr(calc)	ppm	M	Score	Expect	Rank	U	Peptide
5		305.6824	609.3502	609.3486	2.70	0	24	0.0038	1	U	K.TPPAPK.T
34	1	336.2322	670.4498	670.4490	1.28	1	25	0.003	1	U	K.KVAVVR.T
45		340.6774	679.3402	679.3402	0.10	0	7	0.2	1	U	K.HQPGGGK.V
62	1	357.7293	713.4440	713.4436	0.69	0	19	0.011	1	U	K.VQIINK.K
65		358.2212	714.4278	714.4276	0.39	0	28	0.0016	1	U	K.VQIINK.K + Deamidated (NQ)
66		359.1860	716.3574	716.3565	1.27	0	23	0.0056	1	U	K.GQANATR.I
71		363.2016	724.3886	724.3868	2.58	0	32	0.00059	1	U	R.GAAPPGQK.G
80		378.2243	754.4340	754.4337	0.44	1	25	0.003	1	U	K.KIETHK.L
101	2	397.2140	792.4134	792.4130	0.57	1	41	8.6e-05	1	U	K.LDFKDR.V
150		422.2691	842.5236	842.5225	1.34	1	25	0.003	1	U	K.VQIINKK.L + Deamidated (NQ)
176	1	431.2380	860.4614	860.4603	1.30	0	30	0.0009	1	U	K.IGSTENLK.H
178	1	431.7297	861.4448	861.4443	0.59	0	47	2e-05	1	U	K.IGSTENLK.H + Deamidated (NQ)
182		433.7345	865.4544	865.4545	-0.076	1	38	0.00016	1	U	K.SEKLDFK.D
311		489.7715	977.5284	977.5294	-0.98	1	12	0.057	1	U	K.LTFRENAK.A
312		326.8506	977.5300	977.5294	0.58	1	1	0.82	1	U	K.LTFRENAK.A
315		490.2636	978.5126	978.5134	-0.78	1	17	0.021	1	U	K.LTFRENAK.A + Deamidated (NQ)
316		327.1789	978.5149	978.5134	1.49	1	3	0.5	1	U	K.LTFRENAK.A + Deamidated (NQ)
335	3	498.7531	995.4916	995.4924	-0.73	0	50	1.1e-05	1	U	K.TPPSSGEPPK.S
347		502.2736	1002.5326	1002.5346	-1.91	0	59	1.2e-06	1	U	K.LDLSNVQSK.C
352		502.7653	1003.5160	1003.5186	-2.51	0	35	0.00029	1	U	K.LDLSNVQSK.C + Deamidated (NQ)
454	5	533.7966	1065.5786	1065.5819	-3.02	0	48	1.6e-05	1	U	R.TPSLPTPPTR.E
459		356.2011	1065.5815	1065.5819	-0.37	0	12	0.062	1	U	R.TPSLPTPPTR.E

Query	Dupes	Observed	Mr(expt)	Mr(calc)	ppm	M	Score	Expect	Rank	U	Peptide
471		538.8005	1075.5864	1075.5873	-0.80	1	5	0.35	1	U	K.SKIGSTENLK.H
472		359.5371	1075.5895	1075.5873	2.01	1	12	0.059	1	U	K.SKIGSTENLK.H
519	2	551.2806	1100.5466	1100.5462	0.39	0	44	4.4e-05	1	U	K.SPVVSGDTSPR.H
571		563.8197	1125.6248	1125.6254	-0.51	1	5	0.34	1	U	K.GQANATRIPAK.T
572		376.2162	1125.6268	1125.6254	1.19	1	7	0.19	1	U	K.GQANATRIPAK.T
575		564.3112	1126.6078	1126.6094	-1.41	1	4	0.38	1	U	K.GQANATRIPAK.T + Deamidated (NQ)
576		376.5441	1126.6105	1126.6094	0.92	1	11	0.075	1	U	K.GQANATRIPAK.T + Deamidated (NQ)
584	1	566.3204	1130.6262	1130.6295	-2.89	1	53	5.5e-06	1	U	K.KLDLSNVQSK.C
587	2	377.8837	1130.6293	1130.6295	-0.22	1	30	0.001	1	U	K.KLDLSNVQSK.C
591	1	566.7849	1131.5552	1131.5560	-0.69	0	23	0.0048	1	U	K.TDHGAEIVYK.S
592		378.1926	1131.5560	1131.5560	-0.051	0	14	0.039	1	U	K.TDHGAEIVYK.S
596	1	566.8138	1131.6130	1131.6135	-0.42	1	58	1.6e-06	1	U	K.KLDLSNVQSK.C + Deamidated (NQ)
598	1	378.2122	1131.6148	1131.6135	1.10	1	25	0.0033	1	U	K.KLDLSNVQSK.C + Deamidated (NQ)
954	1	655.3631	1308.7116	1308.7112	0.38	0	38	0.00017	1	U	R.LQTAPVPMPDLK.N
955		655.3638	1308.7130	1308.7150	-1.49	1	8	0.15	1	U	R.SRTPSLPTPPTR.E
956	1	437.2456	1308.7150	1308.7150	-0.018	1	13	0.045	1	U	R.SRTPSLPTPPTR.E
987	5	663.3600	1324.7054	1324.7061	-0.47	0	53	5.4e-06	1	U	R.LQTAPVPMPDLK.N + Oxidation (M)
994	1	666.3506	1330.6866	1330.6881	-1.09	1	46	2.6e-05	1	U	K.AKTDHGAEIVYK.S
998	1	444.5701	1330.6885	1330.6881	0.28	1	59	1.2e-06	1	U	K.AKTDHGAEIVYK.S
999	1	333.6797	1330.6897	1330.6881	1.20	1	22	0.0059	1	U	K.AKTDHGAEIVYK.S
1082	2	697.3202	1392.6258	1392.6270	-0.82	0	59	1.3e-06	1	U	R.SGYSSPGSPGTPGSR.S
1103	1	471.2317	1410.6733	1410.6739	-0.46	1	16	0.026	1	U	K.TPPSSGEPPKSGDR.S
1120	1	710.8925	1419.7704	1419.7722	-1.21	1	43	4.6e-05	1	U	R.TPSLPTPPTREPK.K
1122	2	474.2643	1419.7711	1419.7722	-0.77	1	28	0.0017	1	U	R.TPSLPTPPTREPK.K
1235		508.2699	1521.7879	1521.7899	-1.36	1	11	0.085	1	U	K.IGSTENLKHQPGGGK.V
1238		508.5981	1522.7725	1522.7740	-0.97	1	9	0.14	1	U	K.IGSTENLKHQPGGGK.V + Deamidated (NQ)
1284		523.6196	1567.8370	1567.8392	-1.42	1	7	0.18	1	U	K.SRLQTAPVPMPDLK.N + Oxidation (M)
1301	6	526.9448	1577.8126	1577.8162	-2.28	0	36	0.00025	1	U	K.IGSLDNITHVPGGGNK.K
1302	2	789.9138	1577.8130	1577.8162	-1.98	0	36	0.00024	1	U	K.IGSLDNITHVPGGGNK.K
1309		790.4034	1578.7922	1578.8002	-5.03	0	32	0.00065	1	U	K.IGSLDNITHVPGGGNK.K + Deamidated (NQ)
1310		790.4059	1578.7972	1578.8002	-1.86	0	14	0.042	1	U	K.IGSLDNITHVPGGGNK.K + Deamidated (NQ)
1311	1	527.2733	1578.7981	1578.8002	-1.34	0	53	5e-06	1	U	K.IGSLDNITHVPGGGNK.K + Deamidated (NQ)
1421		556.3107	1665.9103	1665.9124	-1.26	1	13	0.055	1	U	R.LQTAPVPMPDLKNVK.S + Oxidation (M)
1480	1	853.9611	1705.9076	1705.9111	-2.04	1	20	0.0095	1	U	K.IGSLDNITHVPGGGNKK.I
1482	5	569.6433	1705.9081	1705.9111	-1.79	1	33	0.00052	1	U	K.IGSLDNITHVPGGGNKK.I
1486	5	427.4846	1705.9093	1705.9111	-1.08	1	22	0.0065	1	U	K.IGSLDNITHVPGGGNKK.I
1680		479.9979	1915.9625	1915.9687	-3.22	0	6	0.26	1	U	K.CGSLGNIHHKPGGGQVEVK.S
1760	1	652.3244	1953.9514	1953.9531	-0.90	0	34	0.00038	1	U	K.STPTAEDVTAPLVDEGAPGK.Q
1763	4	977.9835	1953.9524	1953.9531	-0.35	0	56	2.6e-06	1	U	K.STPTAEDVTAPLVDEGAPGK.Q
1832	3	495.7783	1979.0841	1979.0840	0.033	0	36	0.00025	1	U	K.HVPGGGSVQIVYKPVDLSK.V
1833	8	660.7021	1979.0845	1979.0840	0.22	0	36	0.00028	1	U	K.HVPGGGSVQIVYKPVDLSK.V

2 **ni|1000000007|** 76 Black 5->3 Frame 2 PrpSheep-Hamster

10 per page 1

Not what you expected? Try **the select summary**.

Mascot: http://www.matrixscience.com/
