# Supplementary material for: Fit-for-purpose curated database application in mass spectrometry-based targeted protein identification and validation
Source: BMC Res Notes. 2014 Jul 10;7:444. doi: 10.1186/1756-0500-7-444 (PMC4102332; doi:10.1186/1756-0500-7-444)
Supplement: Additional file 6 — Sh-Ha Chimeric PrP-NCBInr DB search. [file 1756-0500-7-444-S6.pdf]

MASCOT Search Results

User : keding
E-mail : chengkeding@yahoo.com
Search title : flagellin
MS data file : C:\Xcalibur\data\20120119-001-0023-00866-prp\20120119-007-20120118-18.RAW
Database : NCBInr 20120121 (17,072,391 sequences; 5,860,213,228 residues)
Taxonomy : Mammalia (mammals) (1,041,094 sequences)
Timestamp : 24 Jan 2012 at 15:40:48 GMT

Not what you expected? Try **the select summary**.

Search parameters
Score distribution
Legend

Protein Family Summary

Significance threshold p< 0.05 Max. number of families AUTO
Ions score or expect cut-off 0 Dendrograms cut at 0
Preferred taxonomy All entries

Protein families 1–10 (out of 42)

10 per page 1 2 3 4 5 Next

1 **gi|359545611** 4987 Chain A, Structure Of The Chr Of The Prion Protein In Dpc Micelles

		Score	Mass	Matches	Sequences	emPAI
1.1	gi|359545611<br>Chain A, Structure Of The Chr Of The Prion Protein In Dpc Micelles	4987	2490	103 (100)	1 (1)	39009.33

103 peptide matches (21 non-duplicate, 82 duplicate)

Query	Dupes	Observed	Mr(expt)	Mr(calc)	ppm	M	Score	Expect	Rank	U	Peptide
2054		591.5416	2362.1373	2362.1344	1.22	0	64	3.4e-05	1	U	K.HMAGAAAAGAVVGGLGGYMLGSAMSR.-
2059	16	788.3868	2362.1386	2362.1344	1.76	0	108	3.5e-10	1	U	K.HMAGAAAAGAVVGGLGGYMLGSAMSR.-
2062	1	1182.0770	2362.1394	2362.1344	2.13	0	154	2.7e-14	1	U	K.HMAGAAAAGAVVGGLGGYMLGSAMSR.-
2090	17	793.7181	2378.1325	2378.1293	1.32	0	83	1.2e-07	1	U	K.HMAGAAAAGAVVGGLGGYMLGSAMSR.- + Oxidation (M)
2097		595.5406	2378.1333	2378.1293	1.67	0	47	0.0014	1	U	K.HMAGAAAAGAVVGGLGGYMLGSAMSR.- + Oxidation (M)
2099	4	793.7184	2378.1334	2378.1293	1.70	0	97	4.3e-09	1	U	K.HMAGAAAAGAVVGGLGGYMLGSAMSR.- + Oxidation (M)
2100	2	1190.0740	2378.1334	2378.1293	1.73	0	132	4.7e-11	1	U	K.HMAGAAAAGAVVGGLGGYMLGSAMSR.- + Oxidation (M)
2101		1190.0740	2378.1334	2378.1293	1.73	0	123	2.7e-11	1	U	K.HMAGAAAAGAVVGGLGGYMLGSAMSR.- + Oxidation (M)
2111		595.5412	2378.1357	2378.1293	2.68	0	18	0.11	2	U	K.HMAGAAAAGAVVGGLGGYMLGSAMSR.- + Oxidation (M)
2112	2	793.7194	2378.1364	2378.1293	2.96	0	89	4.2e-08	1	U	K.HMAGAAAAGAVVGGLGGYMLGSAMSR.- + Oxidation (M)
2113		1190.0760	2378.1374	2378.1293	3.41	0	129	3.5e-12	1	U	K.HMAGAAAAGAVVGGLGGYMLGSAMSR.- + Oxidation (M)
2132	1	799.0500	2394.1282	2394.1243	1.64	0	126	2.1e-11	1	U	K.HMAGAAAAGAVVGGLGGYMLGSAMSR.- + 2 Oxidation (M)
2137		599.5395	2394.1289	2394.1243	1.94	0	63	4.1e-05	1	U	K.HMAGAAAAGAVVGGLGGYMLGSAMSR.- + 2 Oxidation (M)
2140		1198.0720	2394.1294	2394.1243	2.17	0	117	6.1e-11	1	U	K.HMAGAAAAGAVVGGLGGYMLGSAMSR.- + 2 Oxidation (M)
2143	1	799.0507	2394.1303	2394.1243	2.51	0	111	4.1e-10	1	U	K.HMAGAAAAGAVVGGLGGYMLGSAMSR.- + 2 Oxidation (M)
2144		599.5399	2394.1305	2394.1243	2.61	0	31	0.019	1	U	K.HMAGAAAAGAVVGGLGGYMLGSAMSR.- + 2 Oxidation (M)
2147		1198.0730	2394.1314	2394.1243	3.01	0	132	7.3e-12	1	U	K.HMAGAAAAGAVVGGLGGYMLGSAMSR.- + 2 Oxidation (M)
2148		1198.0730	2394.1314	2394.1243	3.01	0	138	1.7e-12	1	U	K.HMAGAAAAGAVVGGLGGYMLGSAMSR.- + 2 Oxidation (M)
2149	20	799.0511	2394.1315	2394.1243	3.02	0	110	4.8e-10	1	U	K.HMAGAAAAGAVVGGLGGYMLGSAMSR.- + 2 Oxidation (M)
2184		1206.0690	2410.1234	2410.1192	1.77	0	86	4.2e-08	1	U	K.HMAGAAAAGAVVGGLGGYMLGSAMSR.- + 3 Oxidation (M)
2186	18	804.3820	2410.1242	2410.1192	2.08	0	126	2.4e-11	1	U	K.HMAGAAAAGAVVGGLGGYMLGSAMSR.- + 3 Oxidation (M)

2

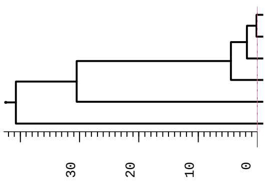

1	gi|4406457	913	prion protein [Addax nasomaculatus]
5	gi|115361493	444	prion protein [Ovis aries]
6	gi|163954796	359	prion protein [Ovis aries]
4	gi|63253843	452	prion protein [Ovis aries]
2	gi|27733816	820	prion protein [Galeopterus variegatus]
3	gi|191432	459	PrP 27-30 protein [Mesocricetus auratus]

Threshold (**0**): 0

		Score	Mass	Matches	Sequences	emPAI
2.1	gi|4406457<br>prion protein [Addax nasomaculatus]	913	21308	78 (60)	7 (6)	2.73
2.2	gi|27733816<br>prion protein [Galeopterus variegatus]	820	26720	59 (50)	6 (5)	1.28
2.3	gi|191432<br>PrP 27-30 protein [Mesocricetus auratus]<br>**1 sameset of gi|191432**	459	16490	37 (29)	6 (5)	4.35
2.4	gi|63253843<br>prion protein [Ovis aries]	452	13298	64 (38)	6 (5)	5.22
2.5	gi|115361493	444	27883	55 (34)	9 (5)	1.21

		Score	Mass	Matches	Sequences	emPAI
	prion protein [Ovis aries]					
☑ 2.6	gi|163954796	359	15203	31 (24)	5 (4)	1.74
	prion protein [Ovis aries]					

▶ **1 sameset of gi|163954796**

▼ **107 peptide matches (29 non-duplicate, 78 duplicate)**

Query	Dupes	Observed	Mr(expt)	Mr(calc)	ppm	M	Score	Expect	Rank	U	1	2	3	4	5	6	Peptide
391		508.7719	1015.5292	1015.5298	-0.59	0	12	0.33	▶1		■	■	■	■	■	■	K.qHTVTTTTK.G
398		513.2664	1024.5182	1024.5302	-11.6	0	29	0.091	▶1	U					■		K.HHTVTTTTK.G
415	▶18	522.7407	1043.4668	1043.4672	-0.33	0	57	0.00014	▶1		■			■	■		R.ESQAYYQR
432		523.2330	1044.4514	1044.4512	0.24	0	35	0.031	▶1		■			■	■		R.ESQAYYQR + Deamidated (NQ)
518		543.2533	1084.4920	1084.4937	-1.56	0	23	0.025	▶1	U		■					K.EQQAYYQR.G
520		543.7463	1085.4780	1085.4778	0.27	0	37	0.083	▶1	U		■					K.EQQAYYQR.G + Deamidated (NQ)
523	▶24	545.2558	1088.4970	1088.4999	-2.64	0	58	0.00013	▶1		■	■					R.YPGQGSPGGNR.Y
548	▶1	545.7485	1089.4824	1089.4839	-1.36	0	46	0.0013	▶1		■	■					R.YPGQGSPGGNR.Y + Deamidated (NQ)
567	▶11	551.7697	1101.5248	1101.5243	0.48	0	46	0.02	▶1	U				■			R.YPNQVYYR.L
578	▶1	552.2625	1102.5104	1102.5083	1.92	0	38	0.029	▶1	U				■			R.YPNQVYYR.L + Deamidated (NQ)
671	▶23	577.2727	1152.5308	1152.5299	0.84	0	70	8.5e-05	▶1		■	■	■	■	■	■	K.GENFTETDIK.I
689		577.7654	1153.5162	1153.5139	2.05	0	48	0.0074	▶1		■	■	■	■	■	■	K.GENFTETDIK.I + Deamidated (NQ)
1149		723.8297	1445.6448	1445.6760	-21.5	1	33	0.0082	▶1	U	■						-.GGSRYPGQGSPGGNR.Y
1239		761.8608	1521.7070	1521.7068	0.15	0	28	0.035	▶1	U						■	R.VVEQMCITHYQR.E + Oxidation (M)
1519		561.6074	1681.8004	1681.7981	1.34	1	9	0.52	▶1		■	■	■	■	■	■	K.GENFTETDIKIMER.V
1534		566.9387	1697.7943	1697.7930	0.73	1	6	1.2	▶2		■	■	■	■	■	■	K.GENFTETDIKIMER.V + Oxidation (M)
1535		849.9047	1697.7948	1697.7930	1.07	1	45	0.00034	▶1		■	■	■	■	■	■	K.GENFTETDIKIMER.V + Oxidation (M)
1655		607.6444	1819.9114	1819.9078	1.98	0	8	1.2	▶2	U			■				-.GQGGGTHNQWNKPSKPK.T
1658		607.9708	1820.8906	1820.8918	-0.67	0	31	0.0078	▶1	U			■				-.GQGGGTHNQWNKPSKPK.T + Deamidated (NQ)
1897		717.6898	2150.0476	2150.0492	-0.74	1	21	0.042	▶1		■	■	■	■	■	■	K.qHTVTTTTKGENFTETDIK.I
2435		879.1640	3512.6269	3512.6572	-8.62	0	16	0.094	▶1	U					■		R.YPNQVYYRPVDQYSNQNNFVHDCVNIPVK.X
2439		888.1699	3548.6505	3548.6129	10.6	0	8	0.53	▶1	U					■		R.YPNQVYYRPVDQYSNQNNFVHDCVNIMVK.X + 2 Deamidated (
2440		1189.2230	3564.6472	3564.6078	11.0	0	28	0.06	▶1	U					■		R.YPNQVYYRPVDQYSNQNNFVHDCVNIMVK.X + 2 Deamidated
2445		901.6812	3602.6957	3602.6677	7.76	0	9	0.46	▶1	U					■		R.YPNQVYYRPVDQYSNQNNFVHDCVNIWVK.X + Deamidated (NQ
2490		978.4406	3909.7333	3909.7266	1.71	0	66	3.5e-05	▶1	U			■				K.HMAGAAAAGAVVGGLGGYMLGSAMSRPMMHFGNDWEDR.Y + 2 Ox
2492		982.4401	3925.7313	3925.7215	2.49	0	34	0.0039	▶1	U			■				K.HMAGAAAAGAVVGGLGGYMLGSAMSRPMMHFGNDWEDR.Y + 3 Ox
2495		986.4385	3941.7249	3941.7164	2.14	0	39	0.0011	▶1	U			■				K.HMAGAAAAGAVVGGLGGYMLGSAMSRPMMHFGNDWEDR.Y + 4 O
2496		986.4386	3941.7253	3941.7164	2.25	0	27	0.0054	▶1	U			■				K.HMAGAAAAGAVVGGLGGYMLGSAMSRPMMHFGNDWEDR.Y + 4 O
2501		990.4383	3957.7241	3957.7114	3.22	0	48	0.00019	▶1	U			■				K.HMAGAAAAGAVVGGLGGYMLGSAMSRPMMHFGNDWEDR.Y + 5 O

▶ **34 subsets and intersections (514 subset proteins in total)**

▶3	gi|435476	340	cytokeratin 9 [Homo sapiens]
▶4	gi|136429	168	RecName: Full=Trypsin; Flags: Precursor
▶5	gi|11935049	167	keratin 1 [Homo sapiens]
▶6	gi|93279106	118	Chain E, Architecture Of Mammalian Fatty Acid Synthase
▶7	gi|125154	102	RecName: Full=Adenylate kinase 2, mitochondrial; Short=AK 2; AltName: Full=ATP-AMP transp...
▶8	gi|297303011	100	PREDICTED: NADH-quinone oxidoreductase subunit B-like, partial [Macaca mulatta]
▶9	gi|291410763	85	PREDICTED: histone cluster 1, H2ag-like [Oryctolagus cuniculus]
▶10	gi|49868	71	put. beta-actin (aa 27-375) [Mus musculus]

10 per page 1 2 3 4 5 Next

Not what you expected? Try **the select summary**.

Mascot: http://www.matrixscience.com/
