# Supplementary material for: Fit-for-purpose curated database application in mass spectrometry-based targeted protein identification and validation
Source: BMC Res Notes. 2014 Jul 10;7:444. doi: 10.1186/1756-0500-7-444 (PMC4102332; doi:10.1186/1756-0500-7-444)
Supplement: Additional file 7 — Sh-Ha PrP-custom DB search. [file 1756-0500-7-444-S7.pdf]

MASCOT Search Results

User : keding
E-mail : chengkeding@yahoo.com
Search title : flagellin
MS data file : C:\Xcalibur\data\20120119-001-0023-00866-prp\20120119-007-20120118-18.RAW
Database : CustomDB (15 sequences; 5,291 residues)
Taxonomy : Mammalia (mammals) (15 sequences)
Timestamp : 24 Jan 2012 at 15:45:57 GMT
Warning : No taxonomy indexes for CustomDB, taxonomy 'Mammalia (mammals)' ignored. Searching all entries in CustomDB

Not what you expected? Try **the select summary**.

Search parameters

Score distribution

Legend

Protein Family Summary

Significance threshold p<	0.05	Max. number of families	AUTO
Ions score or expect cut-off	0	Dendrograms cut at	0

Protein family 1 (out of 1)

10 per page 1

1 **gi|1000000007** 3857 Black 5->3 Frame 2 PrpSheep-Hamster

		Score	Mass	Matches	Sequences	emPAI
**1.1**	**gi|1000000007**<br>Black 5->3 Frame 2 PrpSheep-Hamster	3857	24501	156 (148)	9 (8)	15.62

156 peptide matches (29 non-duplicate, 127 duplicate)

Query	Dupes	Observed	Mr(expt)	Mr(calc)	ppm	M	Score	Expect	Rank	U	Peptide
391		508.7719	1015.5292	1015.5298	-0.59	0	12	0.064	1	U	K.QHTVTTTTK.G
415	18	522.7407	1043.4668	1043.4672	-0.33	0	57	2e-06	1	U	R.ESQAYYQR.G
432		523.2330	1044.4514	1044.4512	0.24	0	35	0.00031	1	U	R.ESQAYYQR.G + Deamidated (NQ)
523	24	545.2558	1088.4970	1088.4999	-2.64	0	58	1.5e-06	1	U	R.YPGQGSPGGNR.Y
548	1	545.7485	1089.4824	1089.4839	-1.36	0	46	2.4e-05	1	U	R.YPGQGSPGGNR.Y + Deamidated (NQ)
671	23	577.2727	1152.5308	1152.5299	0.84	0	70	1.1e-07	1	U	K.GENFTETDIK.I
689		577.7654	1153.5162	1153.5139	2.05	0	48	1.7e-05	1	U	K.GENFTETDIK.I + Deamidated (NQ)
952		688.8270	1375.6394	1375.6415	-1.50	1	53	5e-06	1	U	-.MRYPGQGSPGGNR.Y
953	1	459.5543	1375.6411	1375.6415	-0.32	1	33	0.00046	1	U	-.MRYPGQGSPGGNR.Y
957		459.8773	1376.6101	1376.6255	-11.2	1	6	0.23	1	U	-.MRYPGQGSPGGNR.Y + Deamidated (NQ)
982	23	689.8237	1377.6328	1377.6313	1.11	0	54	3.7e-06	1	U	R.LIHFGNDYEDR.Y
995	32	460.2184	1377.6334	1377.6313	1.49	0	45	3e-05	1	U	R.LIHFGNDYEDR.Y
1016	1	690.3167	1378.6188	1378.6153	2.55	0	46	2.3e-05	1	U	R.LIHFGNDYEDR.Y + Deamidated (NQ)
1018	1	460.5471	1378.6195	1378.6153	3.01	0	42	6.1e-05	1	U	R.LIHFGNDYEDR.Y + Deamidated (NQ)
1041	1	696.8248	1391.6350	1391.6364	-0.99	1	54	4.4e-06	1	U	-.MRYPGQGSPGGNR.Y + Oxidation (M)
1043	2	464.8859	1391.6359	1391.6364	-0.40	1	33	0.00054	1	U	-.MRYPGQGSPGGNR.Y + Oxidation (M)
1045		697.3150	1392.6154	1392.6204	-3.58	1	50	9.6e-06	1	U	-.MRYPGQGSPGGNR.Y + Deamidated (NQ); Oxidation (M)
1046		697.3163	1392.6180	1392.6204	-1.72	1	47	2.1e-05	1	U	-.MRYPGQGSPGGNR.Y + Deamidated (NQ); Oxidation (M)
1047		465.2141	1392.6205	1392.6204	0.024	1	4	0.38	1	U	-.MRYPGQGSPGGNR.Y + Deamidated (NQ); Oxidation (M)
1048		465.2143	1392.6211	1392.6204	0.46	1	16	0.022	1	U	-.MRYPGQGSPGGNR.Y + Deamidated (NQ); Oxidation (M)
1519		561.6074	1681.8004	1681.7981	1.34	1	9	0.12	1	U	K.GENFTETDIKIMER.V
1534		566.9387	1697.7943	1697.7930	0.73	1	6	0.25	1	U	K.GENFTETDIKIMER.V + Oxidation (M)
1535		849.9047	1697.7948	1697.7930	1.07	1	45	3e-05	1	U	K.GENFTETDIKIMER.V + Oxidation (M)
1897		717.6898	2150.0476	2150.0492	-0.74	1	21	0.0086	1	U	K.QHTVTTTTKGENFTETDIK.I
2490		978.4406	3909.7333	3909.7266	1.71	0	66	2.5e-07	1	U	K.HMAGAAAAGAVVGGLGGYMLGSAMSRPMMHFGNDWEDR.Y + 2 Oxidation (M)
2492		982.4401	3925.7313	3925.7215	2.49	0	34	0.00038	1	U	K.HMAGAAAAGAVVGGLGGYMLGSAMSRPMMHFGNDWEDR.Y + 3 Oxidation (M)
2495		986.4385	3941.7249	3941.7164	2.14	0	39	0.00014	1	U	K.HMAGAAAAGAVVGGLGGYMLGSAMSRPMMHFGNDWEDR.Y + 4 Oxidation (M)
2496		986.4386	3941.7253	3941.7164	2.25	0	27	0.0019	1	U	K.HMAGAAAAGAVVGGLGGYMLGSAMSRPMMHFGNDWEDR.Y + 4 Oxidation (M)
2501		990.4383	3957.7241	3957.7114	3.22	0	48	1.4e-05	1	U	K.HMAGAAAAGAVVGGLGGYMLGSAMSRPMMHFGNDWEDR.Y + 5 Oxidation (M)

10 per page 1

Not what you expected? Try **the select summary**.

Mascot: http://www.matrixscience.com/
