# Supplementary material for: Fit-for-purpose curated database application in mass spectrometry-based targeted protein identification and validation
Source: BMC Res Notes. 2014 Jul 10;7:444. doi: 10.1186/1756-0500-7-444 (PMC4102332; doi:10.1186/1756-0500-7-444)
Supplement: Additional file 8 — H37-NCBInr DB search. [file 1756-0500-7-444-S8.pdf]

MASCOT Search Results

User : keding
E-mail : chengkeding@yahoo.com
Search title : flagellin
MS data file : C:\Xcalibur\data\20111209-004-0031-00787\20111209-026-E20506.RAW
Database : NCBInr 20111206 (16,392,747 sequences; 5,641,810,382 residues)
Taxonomy : Bacteria (Eubacteria) (9,447,410 sequences)
Timestamp : 17 Dec 2011 at 16:09:36 GMT

Not what you expected? Try **the select summary**.

- **Search parameters**
- **Score distribution**
- **Legend**

Protein Family Summary

Significance threshold p< 0.05 Max. number of families AUTO
Ions score or expect cut-off 0 Dendrograms cut at 0
Preferred taxonomy All entries

Protein families 1–10 (out of 60)

10 per page 1 2 3 4 5 6 Next

1

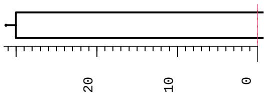

1	gi|301645557	18862	bacterial flagellin [Escherichia coli MS 146-1]
2	gi|121144335	1176	FliA55 [Escherichia coli]

Threshold (**0**): 0

		Score	Mass	Matches	Sequences	emPAI
1.1	gi|301645557<br>bacterial flagellin [Escherichia coli MS 146-1]	18862	58209	436 (403)	31 (26)	10.90
1.2	gi|121144335<br>FliA55 [Escherichia coli]	1176	62285	35 (26)	9 (7)	1.16

439 peptide matches (60 non-duplicate, 379 duplicate)

Query	Dupes	Observed	Mr(expt)	Mr(calc)	ppm	M	Score	Expect	Rank	U	1	2	Peptide
27	3	316.6888	631.3630	631.3653	-3.58	0	35	0.58	1		■	■	R.LSSGLR.I
121		355.1966	708.3786	708.3806	-2.80	0	14	0.88	1		■	■	R.FTSNIK.G
166	1	382.2123	762.4100	762.4123	-2.98	0	36	1.4	3	U	■		K.IDSSTLK.L
412	1	446.2596	890.5046	890.5073	-2.95	1	34	1.5	5	U	■		K.KIDSSTLK.L
502	2	473.2581	944.5016	944.5039	-2.41	0	74	0.00067	1	U	■		R.SSLGAIQNR.L
515		475.7487	949.4828	949.4869	-4.26	0	47	0.059	1	U	■		K.LTGFNVNGK.A + Deamidated (NQ)
522	2	476.7394	951.4642	951.4662	-2.01	0	37	0.026	1	U	■		K.NVYVDASGK.L
901	2	551.2662	1100.5178	1100.5210	-2.89	0	81	4.6e-05	1		■	■	K.DDAAGQAIANR.F
1145	1	596.3004	1190.5862	1190.5891	-2.39	0	61	0.0088	1	U	■		K.NQSALSSSIER.L
1147		596.7922	1191.5698	1191.5731	-2.73	0	50	0.013	1	U	■		K.NQSALSSSIER.L + Deamidated (NQ)
1390	2	648.3449	1294.6752	1294.6769	-1.25	0	69	0.00042	1	U	■		K.ALYIDSTGNLTK.N
1474		672.8770	1343.7394	1343.7408	-1.04	0	82	5.6e-05	1	U		■	-.SLSLITQNNINK.N
1477	1	673.3677	1344.7208	1344.7249	-2.98	0	62	0.0068	1	U		■	-.SLSLITQNNINK.N + Deamidated (NQ)
1637	34	714.3399	1426.6652	1426.6650	0.17	0	115	2.8e-08	1	U	■		R.IDFDSGMSVTLDK.V
1655	1	720.9112	1439.8078	1439.8096	-1.22	0	102	3.5e-07	1		■	■	K.AQIIQQAGNSVLAK.A
1657	1	721.4020	1440.7894	1440.7936	-2.89	0	96	2.1e-06	1		■	■	K.AQIIQQAGNSVLAK.A + Deamidated (NQ)
1662	4	722.3354	1442.6562	1442.6599	-2.55	0	89	7.3e-06	1	U	■		R.IDFDSGMSVTLDK.V + Oxidation (M)
1693	8	729.8922	1457.7698	1457.7726	-1.85	0	115	3.9e-08	1	U	■		K.ITIDGSAQEVNIAK.D
1736		747.9161	1493.8176	1493.8202	-1.70	0	59	0.0063	1	U	■		K.ANQVPQQVLSLLQG.-
1820		781.4193	1560.8240	1560.8260	-1.26	0	51	0.00082	1		■	■	R.VSGQTQFNGVNVLAK.D
1834	2	781.9105	1561.8064	1561.8100	-2.29	0	71	6.5e-05	1		■	■	R.VSGQTQFNGVNVLAK.D + Deamidated (NQ)
1899	1	836.3788	1670.7430	1670.7457	-1.61	0	102	1.8e-08	1		■	■	R.IQDADYATEVSNMSK.A
1900		836.8699	1671.7252	1671.7298	-2.69	0	72	0.00026	1		■	■	R.IQDADYATEVSNMSK.A + Deamidated (NQ)
1918	54	843.4560	1684.8974	1684.8996	-1.26	0	128	1.5e-09	1	U	■		K.IQVGANDGQTITIDLK.K
1961		844.3766	1686.7386	1686.7407	-1.19	0	97	1.3e-07	1		■	■	R.IQDADYATEVSNMSK.A + Oxidation (M)
2032	35	907.4431	1812.8716	1812.8741	-1.37	0	144	4.1e-11	1	U	■		K.GAAVYAAADGSLTTETTSK.S
2060		605.2985	1812.8737	1812.8741	-0.25	0	44	0.0016	1	U	■		K.GAAVYAAADGSLTTETTSK.S
2121	48	947.4986	1892.9826	1892.9844	-0.90	0	128	3.5e-10	1	U	■		K.STTTNFDAATAVNVLAAVK.D
2147	20	632.0020	1892.9842	1892.9844	-0.099	0	64	0.00016	1	U	■		K.STTTNFDAATAVNVLAAVK.D
2247		1022.0180	2042.0214	2042.0167	2.31	0	80	1.1e-06	1	U	■		K.SEATANPLAALDDAISQIDK.F
2251	4	691.6738	2071.9996	2072.0062	-3.20	0	50	0.00094	1	U	■		K.AYTVVNGAESYAVATNNTVK.T + Deamidated (NQ)
2256	6	1037.0090	2072.0034	2072.0062	-1.33	0	127	1.3e-09	1	U	■		K.AYTVVNGAESYAVATNNTVK.T + Deamidated (NQ)
2263		692.0026	2072.9860	2072.9902	-2.04	0	35	0.011	1	U	■		K.AYTVVNGAESYAVATNNTVK.T + 2 Deamidated (NQ)
2267	4	1037.5020	2072.9894	2072.9902	-0.36	0	114	7e-09	1	U	■		K.AYTVVNGAESYAVATNNTVK.T + 2 Deamidated (NQ)
2274		1043.0670	2084.1194	2084.1225	-1.49	0	111	2.9e-08	1	U	■		M.AQVINTNSLSLITQNNINK.N
2308	13	730.3266	2187.9580	2187.9596	-0.76	0	63	1e-05	1	U	■		K.ATVTETYHEFANGNIYDDK.G + Deamidated (NQ)
2310	1	730.3266	2187.9580	2187.9596	-0.76	0	54	9.1e-05	1	U	■		K.ATVTETYHEFANGNIYDDK.G + Deamidated (NQ)
2313	4	1094.9870	2187.9594	2187.9596	-0.088	0	81	1.1e-06	1	U	■		K.ATVTETYHEFANGNIYDDK.G + Deamidated (NQ)

Query	Dupes	Observed	Mr(expt)	Mr(calc)	ppm	M	Score	Expect	Rank	U	1	2	Peptide
2368	▶1	750.3703	2248.0891	2248.0931	-1.80	0	82	1.9e-06	▶1		■	■	R.LDSAVTNLNNTTTNLSEAQSR.I
2369	▶2	1125.0520	2248.0894	2248.0931	-1.63	0	139	3.6e-12	▶1		■	■	R.LDSAVTNLNNTTTNLSEAQSR.I
2373	▶1	1125.5440	2249.0734	2249.0771	-1.63	0	126	1.5e-10	▶1		■	■	R.LDSAVTNLNNTTTNLSEAQSR.I + Deamidated (NQ)
2421	▶27	1176.0900	2350.1654	2350.1686	-1.34	0	139	1.6e-11	▶1	U	■		K.VNSTVDITGASISAAAMTNELTGK.A
2443	▶22	784.3968	2350.1686	2350.1686	-0.0094	0	78	1.1e-06	▶1	U	■		K.VNSTVDITGASISAAAMTNELTGK.A
2509		1290.1070	2578.1994	2578.1995	-0.0058	0	40	0.0044	▶1	U	■		K.NGSDTLTQATLNDVLTGANSVDDTR.I + Deamidated (NQ)
2510		860.4073	2578.2001	2578.1995	0.24	0	82	2.2e-06	▶1	U	■		K.NGSDTLTQATLNDVLTGANSVDDTR.I + Deamidated (NQ)
2511	▶7	865.0862	2592.2368	2592.2402	-1.34	0	98	4.1e-07	▶1	U	■		R.ELTVQATTGTNSQSDLDSIQDEIK.S
2518	▶6	1297.1270	2592.2394	2592.2402	-0.31	0	44	0.0018	▶1	U	■		R.ELTVQATTGTNSQSDLDSIQDEIK.S
2534		877.0991	2628.2755	2628.2739	0.59	0	79	0.00014	▶1		■	■	R.NANDGISVAQTTEGALSEINNNLQR
2535		877.4255	2629.2547	2629.2579	-1.23	0	81	7.9e-05	▶1		■	■	R.NANDGISVAQTTEGALSEINNNLQR + Deamidated (NQ)
2536		1315.6350	2629.2554	2629.2579	-0.94	0	18	0.18	▶1		■	■	R.NANDGISVAQTTEGALSEINNNLQR + Deamidated (NQ)
2537		1315.6360	2629.2574	2629.2579	-0.18	0	7	0.48	▶1		■	■	R.NANDGISVAQTTEGALSEINNNLQR + Deamidated (NQ)
2538		1315.6360	2629.2574	2629.2579	-0.18	0	7	0.9	▶1		■	■	R.NANDGISVAQTTEGALSEINNNLQR + Deamidated (NQ)
2549	▶4	886.4146	2656.2220	2656.2253	-1.25	0	99	5.4e-09	▶1	U	■		K.ATDANLTTAGFTQGVVDSNGNSTWTK.S + Deamidated (NQ)
2553	▶1	1329.1190	2656.2234	2656.2253	-0.70	0	31	0.0055	▶1	U	■		K.ATDANLTTAGFTQGVVDSNGNSTWTK.S + Deamidated (NQ)
2558	▶2	886.4156	2656.2250	2656.2253	-0.12	0	95	2.1e-08	▶1	U	■		K.ATDANLTTAGFTQGVVDSNGNSTWTK.S + Deamidated (NQ)
2562	▶2	1329.1200	2656.2254	2656.2253	0.057	0	23	0.024	▶1	U	■		K.ATDANLTTAGFTQGVVDSNGNSTWTK.S + Deamidated (NQ)
2563	▶2	1329.1210	2656.2274	2656.2253	0.81	0	25	0.016	▶1	U	■		K.ATDANLTTAGFTQGVVDSNGNSTWTK.S + Deamidated (NQ)
2598	▶12	966.4523	2896.3351	2896.3363	-0.43	0	94	1.4e-08	▶1	U	■		K.SYTFDSTGAAVAGAASSLQGTFGTDTNTAK.I
2607		1449.1770	2896.3394	2896.3363	1.09	0	10	0.88	▶1	U	■		K.SYTFDSTGAAVAGAASSLQGTFGTDTNTAK.I
2643	▶35	979.7844	2936.3314	2936.3312	0.061	0	115	4.4e-10	▶1	U	■		K.DGSTINYTGTGNGLGIAATSAYTYHDSTK.S + Deamidated (NQ)

▶76 subsets and intersections (820 subset proteins in total)

▶2 gi|9256992 529 Chain A, Core Structure Of The Outer Membrane Lipoprotein From Escherichia Coli At 1.9 Angst...

▶3 gi|15799800 466 dihydrolipoamide dehydrogenase [Escherichia coli O157:H7 str. EDL933]

▶4 gi|30062494 185 outer membrane protein A [Shigella flexneri 2a str. 2457T]

▶5

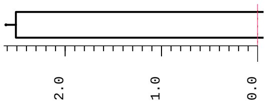

1 gi|15804576 184 50S ribosomal protein L7/L12 [Escherichia coli O157:H7 str. EDL933]

2 gi|152972844 102 50S ribosomal protein L7/L12 [Klebsiella pneumoniae subsp. pneumoniae MGH 78578]

▶6 gi|2624772 175 Chain A, Crystal Structure Of The Asymmetric Chaperonin Complex GroelGROES(ADP)7

▶7 gi|15802055 162 outer membrane protein [Escherichia coli O157:H7 str. EDL933]

▶8 gi|16128108 130 pyruvate dehydrogenase, dihydrolipoyltransacetylase component E2 [Escherichia coli str. K-12 ...

▶9

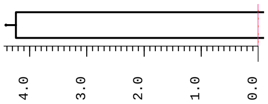

1 gi|1421424 125 Chain O, Comparison Of The Structures Of Wild Type And A N313t Mutant Of Escherichia Coli G...

2 gi|226343955 62 GapA [Escherichia coli]

▶10 gi|15803825 108 30S ribosomal protein S13 [Escherichia coli O157:H7 str. EDL933]

10 per page 1 2 3 4 5 6 Next

Not what you expected? Try the select summary.

Mascot: http://www.matrixscience.com/
