# Supplementary material for: Fit-for-purpose curated database application in mass spectrometry-based targeted protein identification and validation
Source: BMC Res Notes. 2014 Jul 10;7:444. doi: 10.1186/1756-0500-7-444 (PMC4102332; doi:10.1186/1756-0500-7-444)
Supplement: Additional file 9 — H37-E coli-flagellar DB search. [file 1756-0500-7-444-S9.pdf]

MASCOT Search Results

User : keding
E-mail : chengkeding@yahoo.com
Search title : flagellin
MS data file : C:\Xcalibur\data\20111209-004-0031-00787\20111209-026-E20506.RAW
Database : Flagellin_v2 (195 sequences; 91,182 residues)
Taxonomy : Bacteria (Eubacteria) (195 sequences)
Timestamp : 17 Dec 2011 at 17:34:55 GMT

Not what you expected? Try **the select summary**.

Search parameters
Score distribution
Legend

Protein Family Summary

Significance threshold p< 0.05 Max. number of families AUTO
Ions score or expect cut-off 0 Dendrograms cut at 0

Protein families 1–2 (out of 2)

10 per page 1

1

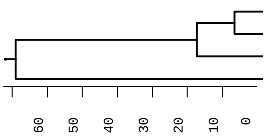

1	**gi|30059906**	29742	H37 [gi|301076183| Hxx] [gi|301645557| Hxx]
4	**gi|40353143**	212	H8
2	**gi|5107845**	1909	H7 [gi|5107857| Hxx]
3	**gi|121144335**	1887	H55

Threshold (**0**): 0

		Score	Mass	Matches	Sequences	emPAI
**1.1**	**gi|30059906**<br>H37 [gi|301076183| Hxx] [gi|301645557| Hxx]	29742	58209	449 (431)	33 (31)	14.66
**1.2**	**gi|5107845**<br>H7 [gi|5107857| Hxx]	1909	56638	40 (34)	11 (10)	1.61
**1.3**	**gi|121144335**<br>H55	1887	62285	42 (33)	11 (9)	1.40
**1.4**	**gi|40353143**<br>H8	212	47290	8 (7)	3 (3)	0.22

457 peptide matches (68 non-duplicate, 389 duplicate)

Query	Dupes	Observed	Mr(expt)	Mr(calc)	ppm	M	Score	Expect	Rank	U	1	2	3	4	Peptide
27	3	316.6888	631.3630	631.3653	-3.58	0	35	0.0026	1		■	■	■	■	R.LSSGLR.I
121		355.1966	708.3786	708.3806	-2.80	0	14	0.25	1		■	■	■		R.FTSNIK.G
159	2	380.6941	759.3736	759.3763	-3.45	0	33	0.0027	1		■	■	■		R.LDEIDR.V
166	2	382.2123	762.4100	762.4123	-2.98	0	36	0.00023	1	U	■				K.IDSSTLK.L
412	1	446.2596	890.5046	890.5073	-2.95	1	34	0.0004	1	U	■				K.KIDSSTLK.L
486	1	467.2346	932.4546	932.4563	-1.77	0	8	0.61	1	U			■		R.SSLGAVQNR.L + 2 Deamidated (NQ)
502	2	473.2581	944.5016	944.5039	-2.41	0	74	1e-07	1		■	■			R.SSLGAIQNR.L
515		475.7487	949.4828	949.4869	-4.26	0	47	1.8e-05	1	U	■				K.LTGFNVNGK.A + Deamidated (NQ)
522	2	476.7394	951.4642	951.4662	-2.01	0	37	0.0002	1	U	■				K.NVYVDASGK.L
795		530.2937	1058.5728	1058.5607	11.4	0	20	0.011	1	U				■	-.LLTQNNLNK.S + 2 Deamidated (NQ)
834		539.2784	1076.5422	1076.5462	-3.65	0	30	0.00094	1	U		■			-.QSALSSSIER.L
901	2	551.2662	1100.5178	1100.5210	-2.89	0	81	6.8e-08	1		■	■	■	■	K.DDAAGQAIANR.F
1145	1	596.3004	1190.5862	1190.5891	-2.39	0	61	3.6e-06	1	U	■				K.NQSALSSSIER.L
1147		596.7922	1191.5698	1191.5731	-2.73	0	50	4.1e-05	1	U	■				K.NQSALSSSIER.L + Deamidated (NQ)
1390	2	648.3449	1294.6752	1294.6769	-1.25	0	69	1.2e-07	1	U	■				K.ALYIDSTGNLTK.N
1474		672.8770	1343.7394	1343.7408	-1.04	0	82	6.3e-09	1	U			■		-.SLSLITQNNINK.N
1477	1	673.3677	1344.7208	1344.7249	-2.98	0	62	6.1e-07	1	U			■		-.SLSLITQNNINK.N + Deamidated (NQ)
1481		674.3489	1346.6832	1346.6929	-7.15	0	2	0.69	1	U			■		-.SLSLITQNNINK.N + 3 Deamidated (NQ)
1637	34	714.3399	1426.6652	1426.6650	0.17	0	115	3.5e-12	1	U	■				R.IDFDSGMSVTLDK.V
1655	1	720.9112	1439.8078	1439.8096	-1.22	0	102	2.8e-10	1		■	■	■		K.AQIIQQAGNSVLAK.A
1657	1	721.4020	1440.7894	1440.7936	-2.89	0	96	1.2e-09	1		■	■	■		K.AQIIQQAGNSVLAK.A + Deamidated (NQ)
1662	5	722.3354	1442.6562	1442.6599	-2.55	0	89	1.1e-09	1	U	■				R.IDFDSGMSVTLDK.V + Oxidation (M)
1693	8	729.8922	1457.7698	1457.7726	-1.85	0	115	5.2e-12	1	U	■				K.ITIDGSAQEVNIAK.D
1701		730.8824	1459.7502	1459.7406	6.63	0	1	1.7	1	U	■				K.ITIDGSAQEVNIAK.D + 2 Deamidated (NQ)
1736		747.9161	1493.8176	1493.8202	-1.70	0	59	6e-06	1	U	■				K.ANQVPQQVLSLLQG.-
1820		781.4193	1560.8240	1560.8260	-1.26	0	51	4e-05	1		■	■	■		R.VSGQTQFNGVNVLAK.D
1834	2	781.9105	1561.8064	1561.8100	-2.29	0	71	3.5e-07	1		■	■	■		R.VSGQTQFNGVNVLAK.D + Deamidated (NQ)
1899	1	836.3788	1670.7430	1670.7457	-1.61	0	102	4.1e-10	1		■	■	■		R.IQDADYATEVSNMSK.A
1900		836.8699	1671.7252	1671.7298	-2.69	0	72	3.8e-07	1		■	■	■		R.IQDADYATEVSNMSK.A + Deamidated (NQ)
1918	54	843.4560	1684.8974	1684.8996	-1.26	0	128	3.9e-13	1	U	■				K.IQVGANDGQTITIDLK.K
1961		844.3766	1686.7386	1686.7407	-1.19	0	97	1.3e-09	1		■	■	■		R.IQDADYATEVSNMSK.A + Oxidation (M)
2032	35	907.4431	1812.8716	1812.8741	-1.37	0	144	3.8e-15	1	U	■				K.GAAVYAAADGSLTTETTSK.S
2060		605.2985	1812.8737	1812.8741	-0.25	0	44	4e-05	1	U	■				K.GAAVYAAADGSLTTETTSK.S
2121	48	947.4986	1892.9826	1892.9844	-0.90	0	128	1.7e-13	1	U	■				K.STTTNFDAATAVNVLAAVK.D

Query	Dupes	Observed	Mr(expt)	Mr(calc)	ppm	M	Score	Expect	Rank	U	1	2	3	4	Peptide
2147	22	632.0020	1892.9842	1892.9844	-0.099	0	64	4.1e-07	1	U	■				K.STTTNFDAATAVNVLAAVK.D
2242		676.0060	2024.9962	2025.0266	-15.0	1	1	0.77	1	U	■				K.ITDTDGKALYIDSTGNLTK.N
2247		1022.0180	2042.0214	2042.0167	2.31	0	80	8.9e-09	1	U	■				K.SEATANPLAALDDAISQIDK.F
2251	4	691.6738	2071.9996	2072.0062	-3.20	0	50	9.4e-06	1	U	■				K.AYTVVNGAESYAVATNNTVK.T + Deamidated (NQ)
2256	6	1037.0090	2072.0034	2072.0062	-1.33	0	127	2.2e-13	1	U	■				K.AYTVVNGAESYAVATNNTVK.T + Deamidated (NQ)
2263		692.0026	2072.9860	2072.9902	-2.04	0	35	0.00031	1	U	■				K.AYTVVNGAESYAVATNNTVK.T + 2 Deamidated (NQ)
2267	4	1037.5020	2072.9894	2072.9902	-0.36	0	114	4.3e-12	1	U	■				K.AYTVVNGAESYAVATNNTVK.T + 2 Deamidated (NQ)
2274		1043.0670	2084.1194	2084.1225	-1.49	0	111	5.5e-11	1	U	■				M.AQVINTNSLSLITQNNINK.N
2308	13	730.3266	2187.9580	2187.9596	-0.76	0	63	5.4e-07	1	U	■				K.ATVTETYHEFANGNIYDDK.G + Deamidated (NQ)
2310	1	730.3266	2187.9580	2187.9596	-0.76	0	54	4e-06	1	U	■				K.ATVTETYHEFANGNIYDDK.G + Deamidated (NQ)
2313	4	1094.9870	2187.9594	2187.9596	-0.088	0	81	7.2e-09	1	U	■				K.ATVTETYHEFANGNIYDDK.G + Deamidated (NQ)
2368	1	750.3703	2248.0891	2248.0931	-1.80	0	82	4e-08	1		■	■	■		R.LDSAVTNLNNTTTNLSEAQSR.I
2369	2	1125.0520	2248.0894	2248.0931	-1.63	0	139	7.8e-14	1		■	■	■		R.LDSAVTNLNNTTTNLSEAQSR.I
2373	1	1125.5440	2249.0734	2249.0771	-1.63	0	126	1.5e-12	1		■	■	■		R.LDSAVTNLNNTTTNLSEAQSR.I + Deamidated (NQ)
2421	27	1176.0900	2350.1654	2350.1686	-1.34	0	139	1.3e-14	1	U	■				K.VNSTVDITGASISAAAMTNELTGK.A
2443	22	784.3968	2350.1686	2350.1686	-0.0094	0	78	1.7e-08	1	U	■				K.VNSTVDITGASISAAAMTNELTGK.A
2509		1290.1070	2578.1994	2578.1995	-0.0058	0	40	0.00011	1	U	■				K.NGSDTLTQATLNDVLTGANSVDDTR.I + Deamidated (NQ)
2510		860.4073	2578.2001	2578.1995	0.24	0	82	6.6e-09	1	U	■				K.NGSDTLTQATLNDVLTGANSVDDTR.I + Deamidated (NQ)
2511	7	865.0862	2592.2368	2592.2402	-1.34	0	98	1.6e-10	1	U	■				R.ELTVQATTGTNSQSDLDSIQDEIK.S
2518	6	1297.1270	2592.2394	2592.2402	-0.31	0	44	4.3e-05	1	U	■				R.ELTVQATTGTNSQSDLDSIQDEIK.S
2533		1315.1440	2628.2734	2628.2739	-0.18	0	3	2.5	1		■	■	■		R.NANDGISVAQTTEGALSEINNNLQR
2534		877.0991	2628.2755	2628.2739	0.59	0	79	5.8e-08	1		■	■	■		R.NANDGISVAQTTEGALSEINNNLQR
2535		877.4255	2629.2547	2629.2579	-1.23	0	81	3.5e-08	1		■	■	■		R.NANDGISVAQTTEGALSEINNNLQR + Deamidated (NQ)
2536		1315.6350	2629.2554	2629.2579	-0.94	0	18	0.077	1		■	■	■		R.NANDGISVAQTTEGALSEINNNLQR + Deamidated (NQ)
2537		1315.6360	2629.2574	2629.2579	-0.18	0	7	0.83	1		■	■	■		R.NANDGISVAQTTEGALSEINNNLQR + Deamidated (NQ)
2538		1315.6360	2629.2574	2629.2579	-0.18	0	7	0.88	1		■	■	■		R.NANDGISVAQTTEGALSEINNNLQR + Deamidated (NQ)
2549	5	886.4146	2656.2220	2656.2253	-1.25	0	99	2.8e-10	1	U	■				K.ATDANLTTAGFTQGVVDSNGNSTWTK.S + Deamidated (NQ)
2553	1	1329.1190	2656.2234	2656.2253	-0.70	0	31	0.0017	1	U	■				K.ATDANLTTAGFTQGVVDSNGNSTWTK.S + Deamidated (NQ)
2558	2	886.4156	2656.2250	2656.2253	-0.12	0	95	7e-10	1	U	■				K.ATDANLTTAGFTQGVVDSNGNSTWTK.S + Deamidated (NQ)
2562	2	1329.1200	2656.2254	2656.2253	0.057	0	23	0.013	1	U	■				K.ATDANLTTAGFTQGVVDSNGNSTWTK.S + Deamidated (NQ)
2563	2	1329.1210	2656.2274	2656.2253	0.81	0	25	0.0076	1	U	■				K.ATDANLTTAGFTQGVVDSNGNSTWTK.S + Deamidated (NQ)
2598	12	966.4523	2896.3351	2896.3363	-0.43	0	94	3.6e-10	1	U	■				K.SYTFDSTGAAVAGAASSLQGTFGTDTNTAK.I
2607	2	1449.1770	2896.3394	2896.3363	1.09	0	10	0.11	1	U	■				K.SYTFDSTGAAVAGAASSLQGTFGTDTNTAK.I
2643	35	979.7844	2936.3314	2936.3312	0.061	0	115	4.6e-12	1	U	■				K.DGSTINYTGTGNGLGIAATSAYTYHDSTK.S + Deamidated (NQ)

55 subsets and intersections (170 subset proteins in total)

2 gi|112820172 18 H21

10 per page 1

Not what you expected? Try the select summary.

Mascot: http://www.matrixscience.com/
